# Geographical and temporal distribution of safeguarding concerns among older adults with dementia in England: a population-based repeated cross-sectional study

**DOI:** 10.1136/bmjopen-2025-110575

**Published:** 2026-08-26

**Authors:** Asri Maharani, Jeremy Dixon, Katarzyna Szulewska, Catherine Robinson, Mark Cooper, Maria Panagioti, Thomas Blakeman, Reena Lasrado

**Affiliations:** 1 Division of Nursing, Midwifery and Social Work, The University of Manchester, Manchester, UK; 2 Centre for Adult Social Care Research (CARE), Cardiff University, Cardiff, UK; 3 Division of Population Health, Health Services Research & Primary Care, University of Manchester, Manchester, UK; 4 National Institute for Health and Care Research (NIHR) Greater Manchester Patient Safety Research Collaboration (GM PSRC), The University of Manchester, Manchester, UK

**Keywords:** Health policy, MENTAL HEALTH, Dementia

## Abstract

**Abstract:**

**Objective:**

To examine the geographical and temporal distribution of safeguarding concerns and enquiries among older adults living with dementia in England, and to assess variation by sex, age and region.

**Design:**

Population-based, repeated cross-sectional study.

**Setting:**

England, using national administrative datasets.

**Participants:**

Older adults living with dementia in England, identified using national dementia prevalence estimates; safeguarding activity was analysed at local authority and regional level.

**Primary and secondary outcome measures:**

Primary outcomes were the number of safeguarding concerns and Section 42 safeguarding enquiries recorded by local authorities. Secondary outcomes included other safeguarding enquiries and variation by age, sex and geographic region.

**Results:**

Safeguarding concerns more than doubled between 2015 and 2022 across the adult population. Among adults with dementia, safeguarding concerns increased by 55.5% between 2015/2016 and 2019/2020, with a temporary decline during the COVID-19 pandemic. Women with dementia, particularly those aged 85 and older, had higher rates of safeguarding concerns and enquiries than men. Spatial analysis revealed substantial variation across England, with higher safeguarding activity in urban and more deprived regions, such as parts of the North West (eg, Manchester, Liverpool), Yorkshire and the Humber, and the Midlands, while lower activity was observed in more rural and less deprived areas such as the North East and South West.

**Conclusions:**

This study provides the first national analysis of safeguarding patterns for people living with dementia in England. The findings found demographic and geographic disparities in safeguarding activity, highlighting the need for more detailed and individual-level data to inform responsive, dementia-sensitive safeguarding policies.

**STRENGTHS AND LIMITATIONS OF THIS STUDY**

- The study uses national routinely collected administrative data covering all local authorities in England, enabling population-level analysis over time.
- Safeguarding activity was analysed using repeated cross-sectional data across multiple years, allowing assessment of temporal trends.
- All analyses were conducted using fully anonymised, aggregate-level data, eliminating risks related to individual identification.
- Differences in dataset structure, coverage and reporting periods required harmonisation, which may have introduced measurement uncertainty.
- Variation in local authority reporting practices and administrative coding may affect completeness and comparability across regions and time.

## Background

 Dementia affects around 55 million people globally, and that figure is expected to triple by 2050.^1^ This figure is significant as older adults living with dementia are at heightened risk of abuse, neglect and exploitation.^2–4^ Such abuse may be psychological, emotional, physical, financial or sexual. People living with dementia may self-neglect, be overmedicated or inappropriately restrained. Furthermore, this population is more likely to experience multiple forms of abuse compared with other older adults.^5^

In recent years, there has been international recognition that more needs to be done to protect older adults from abuse and neglect and that people living with dementia are often at risk of such mistreatment.^6^ The mechanisms through which individuals may be protected from abuse and neglect vary internationally.^7^ Our study focuses on practice in England, in which the process of responding to adult abuse and neglect is referred to as ‘adult safeguarding’. The criteria for safeguarding adults are provided in the Care Act 2014.^8^ Safeguarding duties apply where, “a local authority has reasonable cause to suspect that an adult in its area (whether or not ordinarily resident there)

- has care and support needs (whether or not the authority is meeting those needs)
- is experiencing, or at risk of abuse and neglect, and
- as a result of those needs is unable to protect himself or herself against the abuse or neglect or the risk of it” (Section 42(1)).

Where these conditions are met, the local authority “must make (or cause to be made) whatever enquiries it thinks necessary to enable it to decide whether any action should be taken in the adult’s case…and, if so, what and by whom” (Section 42(2)). Safeguarding concerns can be raised by any professional or member of the public. Once a concern is raised, the local authority has a duty to assess whether the statutory criteria under Section 42 of the Care Act 2014 are met.^8^ If the criteria are met, the concern progresses to a Section 42 Safeguarding Enquiry, which is a formal statutory investigation into the risk of abuse or neglect. If the criteria are not met, the local authority may instead initiate an ‘Other Enquiry’ or take alternative action, such as conducting a needs assessment under the Care Act 2014 or referring the case to another service.^9^ Recent research has provided a conceptual and practice-based understanding of how safeguarding operates for people living with dementia within this legislative framework.^10^

Existing research provides some useful information about the abuse of people living with dementia. A systematic review on 49 studies in Asia, Europe and the USA reported high prevalence of abusive behaviours self-reported by family caregivers of people living with dementia, with overall abuse ranging from 1.6% to 62.3%, psychological abuse from 9.7% to 62.3%, and physical abuse from 1.6% to 18.0%.^2^ These caregiver-reported prevalence estimates were generally higher than those reported in Western countries, where overall abuse ranged from 5.4% to 51.0%, physical abuse from 1.5% to 20.0%, and psychological abuse from 3.5% to 51.0%. The wide range in these estimates reflects heterogeneity across studies included in the review, attributable to differences in definitions of abuse, sampling methods, cultural contexts and the countries and time periods in which studies were conducted.^2^ The majority of the reviewed studies relied solely on reports from family caregivers.^2^ Research studies with family caregivers have identified that instances of abuse and neglect are particularly high where carers are co-residing with the person, possibly due to stress and conflict brought about by the caregiving role.^3 11 12^ Research also indicates that caregiver distress is strongly associated with adverse health outcomes for people living with dementia.^13^

While these studies are useful, research into local authority safeguarding responses to people living with dementia is much more limited. Studies so far have focused on the adult safeguarding coordinators’ perspective on issues of financial abuse^14 15^ and on messages from serious case reviews (now safeguarding adults reviews) as to local authority failures to protect people living with dementia from abuse or neglect.^16^ However, we have not been able to identify any studies which have quantitatively examined adult safeguarding data in relation to people living with dementia. This gap is significant as serious case reviews have identified systemic gaps and poor continuity of care within dementia support services, with people living with dementia often experiencing poor continuity of care.^16^ Moreover, studies not specific to people living with dementia have shown gaps in the practical application and accessibility of safeguarding measures across diverse communities.^17^ Consequently, there is a need to identify patterns of need, abuse or neglect for those living with dementia.^18^

This study aims to identify the area-level distribution of safeguarding among individuals with dementia in England to better understand patterns of reporting and how they vary across location, gender and time period. This allows for the description of safeguarding among older people with dementia to be undertaken across policy-relevant areas such as local authority districts (LADs). The specific objectives of this study are:

1. To analyse the temporal trends (2015–2022) in safeguarding concerns and enquiries in England, including Section 42 Safeguarding Enquiries and ‘Other Enquiries’ (where the adult does not meet the Section 42 criteria, but where the local authority feels it is necessary and proportionate for an enquiry to take place nonetheless).
2. To examine demographic differences in safeguarding concerns reported to the local authority, with a focus on sex and age-related variations among individuals with dementia.
3. To assess the regional distribution of safeguarding concerns and enquiries across LADs in England using the most recent reporting year available from the National Health Service (NHS) Digital Safeguarding Adults Collection at the time of analysis (2021/2022).

## Approach

### Data sources

This study drew on three national data sources: (1) the NHS Digital Safeguarding Adults Collection was used to analyse annual counts of safeguarding concerns and enquiries reported by local authorities in England between 2015 and 2022^19^; (2) NHS Digital Recorded Dementia Diagnoses data (2022)^20^ were used to provide cross-sectional estimates of dementia prevalence by age and sex, which informed proportional allocation of safeguarding activity among people living with dementia and (3) Office for National Statistics (ONS) 2021^21^ population projections were used to estimate age-specific and sex-specific population distributions at local authority level.

The NHS Digital Safeguarding Adults, England dataset is an annual national dataset collection that provides comprehensive insights into safeguarding concerns and enquiries reported by local authorities under the Care Act 2014. It captures data on adults at risk of abuse or neglect, including those subject to formal safeguarding interventions. The dataset includes the total number of safeguarding concerns raised, the number of Section 42 enquiries initiated, and other safeguarding enquiries. Other Safeguarding Enquiries are defined by NHS Digital as enquiries ‘where a concern is raised about a risk of abuse but does not meet the three criteria under Section 42 of the Care Act 2014’.^19^ It provides a demographic breakdown of individuals involved, including age, gender and disability status, as well as information on the types and locations of alleged abuse.

The NHS Digital Recorded Dementia Diagnoses 2022 dataset provides national and regional statistics on the number of individuals diagnosed with dementia in England. Derived from primary care records, this dataset reflects dementia diagnoses recorded within general practitioner (GP) practices that participate in the General Practice Extraction Service for the Quality and Outcomes Framework (QOF) data collection. It includes the total number of recorded dementia diagnoses, along with diagnosis rates at the national, regional and Clinical Commissioning Group (CCG) levels. It provides a demographic breakdown by age group and sex, offering insights into patterns of dementia prevalence across different population groups.

The 2021 ONS Population Projections for Local Authorities provide estimated population figures for local areas in England, based on recent demographic trends. The dataset includes population estimates by age, sex, and geographic region, enabling detailed analysis at the local authority level.

### Measures

Safeguarding concerns and enquiries are recorded in the Safeguarding Adults Collection in the reporting year in which they are first raised or opened. Section 42 enquiries may extend beyond a single reporting year; however, each enquiry is counted only once, in the year it is initiated, and is not re-recorded when concluded.

### Data analysis

Descriptive analyses were used to examine the prevalence and trends of safeguarding concerns and enquiries in England between 2015 and 2022. We obtained the total number of safeguarding concerns and enquiries for individuals with dementia from the NHS Digital Safeguarding Adults dataset. To estimate the number of safeguarding concerns and enquiries by sex and age groups in each region, we used the NHS Digital Recorded Dementia Diagnoses 2022 and the 2021 ONS population projections for local authorities. This approach enabled us to distribute the total reported safeguarding cases proportionally across demographic groups, providing age-specific and sex-specific estimates for each region. Temporal trends were analysed using annual data from 2015 to 2022, whereas geographical variation was examined using data from the most recent reporting year (2021/2022). Finally, we mapped the distribution of (1) total safeguarding concerns; (2) Section 42 Safeguarding Enquiries and (3) Other Safeguarding Enquiries in 314 LADs for all individuals and those with dementia in England for 2021/2022, using STATA V.18.^22^

To enhance transparency, we acknowledge that dementia diagnoses from the QOF register are incomplete and likely underestimate true prevalence. Additionally, data on safeguarding is suppressed when case counts fall below disclosure thresholds, and reporting practices vary among local authorities. To mitigate these issues, we: (a) applied proportional allocation based on national prevalence and population estimates; (b) verified internal consistency by comparing aggregate totals with published NHS Digital reports and (c) undertook sensitivity checks to ensure that proportional distributions aligned with regional prevalence patterns. While these steps cannot fully remove the limitations of the source data, they represent the most robust use of the best-available national datasets to estimate safeguarding patterns among people with dementia.

### Patient and public involvement

Neither patients nor the public were involved in this secondary data analysis.

## Results

### Safeguarding prevalence

**Figure 1:**
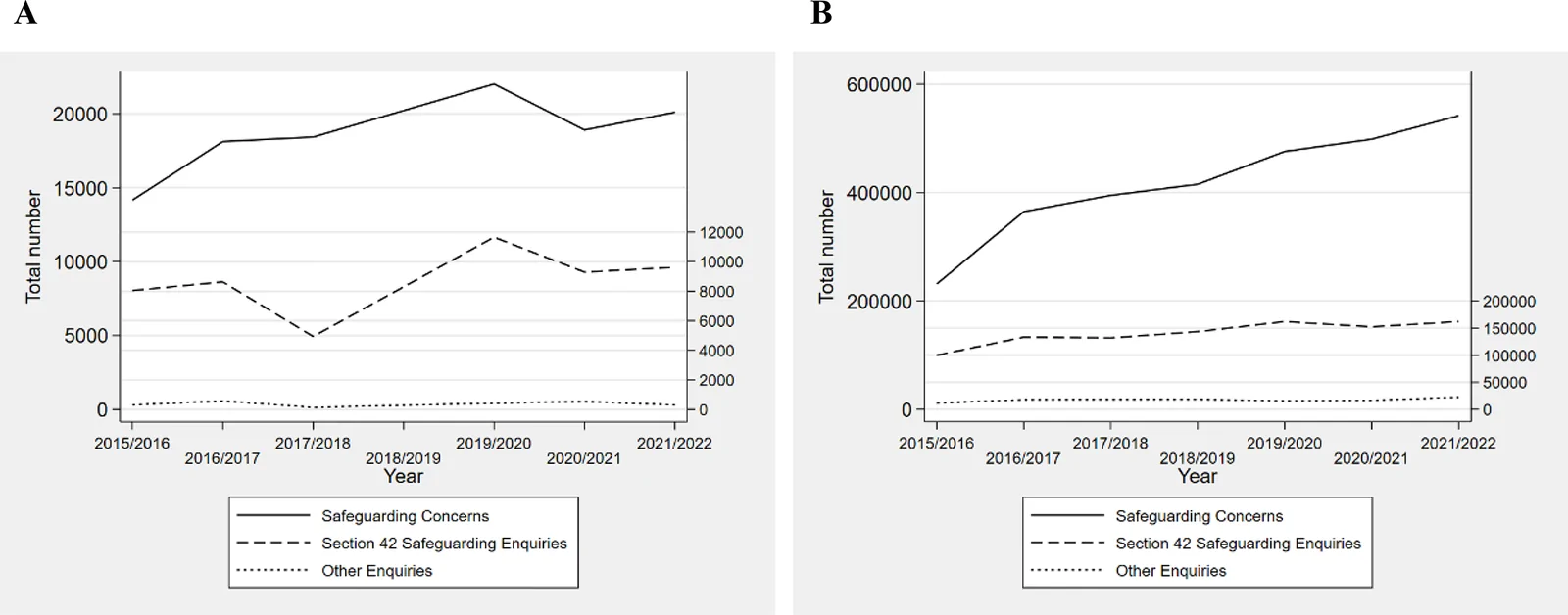
Counts of concerns and enquiries for (**A**) individuals with dementia and (**B**) all individuals 2015–2022. Source: National Health Services (NHS) Digital Safeguarding Adults, England.

Between 2015/2016 and 2021/2022, the dataset captured over 120 000 safeguarding concerns involving individuals living with dementia in England. Focusing on individuals with dementia (figure 1A), safeguarding concerns increased by 55.45%, from 14 165 in 2015/2016 to a peak of 22 020 in 2019/2020, reflecting a gradual rise in reported safeguarding issues for this group. However, a notable decline occurred in 2020/2021, followed by a partial recovery in 2021/2022, although levels remained below the prepandemic peak. The trend in Section 42 Safeguarding Enquiries follows a similar pattern but with greater fluctuation. Enquiries declined sharply between 2016/2017 (8045) and 2017/2018 (4935), before rising again and peaking at 11 655 in 2019/2020. In contrast, other enquiries remained low throughout the period.

Figure 1B illustrates the trends in safeguarding concerns and enquiries for all individuals recorded between 2015 and 2022. Across this period, the NHS Digital Safeguarding Adults dataset captured a total of over 3.3 million safeguarding concerns and more than 1.2 million Section 42 enquiries, providing the overall sample for trend analyses. It shows a steady and substantial increase over the 7-year period. The number of safeguarding concerns rose from 231 220 in 2015/2016 to 541 535 in 2021/2022, representing an increase of 134%. Section 42 safeguarding enquiries increased from 111 460 in 2015/2016 to 177 565 in 2019/2020, declined in 2020/2021 and then rose to 184 515 in 2021/2022. In contrast, ‘Other’ Safeguarding Enquiries remained consistently low, with modest increases over time, but their absolute numbers remained negligible compared with overall safeguarding concerns and Section 42 enquiries.

Online supplemental table 1 shows the estimated sex and age-related distribution of safeguarding concerns among individuals with dementia in England from 2021 to 2022. The total number of safeguarding concerns for males with dementia was 7503, while for females, it was significantly higher at 12 607. Similarly, Section 42 Safeguarding Enquiries were estimated to be more frequent among females (6028 enquiries) compared with males (3587 enquiries). Other Safeguarding Enquiries, which include cases that do not meet the statutory Section 42 threshold, were also estimated to be more common among females (188 cases) than males (112 cases). Age-related trends show that estimated safeguarding concerns increased significantly with age, peaking in the 80–84 (1812 concerns), 85–89 (1713 concerns) and 75–79 (1426 concerns) age groups among males. This pattern was also estimated in females, where the highest number of safeguarding concerns was recorded in the 85–89 (3237 concerns) and 90+ (3205 concerns) age groups, followed by the 80–84 age group (2774 concerns). Safeguarding concerns among individuals under 60 were relatively low compared with older age groups, with approximately 148 cases among males and 141 cases among females.

The number of Section 42 Safeguarding Enquiries was estimated to follow a similar trend, with the highest number of enquiries occurring in the 80–84 (866 enquiries) and 85–89 (819 enquiries) age groups for males. While for females, the corresponding figures for these two age groups were estimated to be significantly higher, at 1326 and 1548 enquiries, respectively. The gender gap in safeguarding cases widens in older age groups, suggesting that older females with dementia may be at higher risk of safeguarding concerns or are more likely to be reported compared with males. The low number of Other Safeguarding Enquiries across all age groups, accounting for less than 1% of total safeguarding concerns, indicates that the majority of concerns either met the statutory threshold for Section 42 enquiries or were resolved through alternative means without a formal enquiry.

**Figure 2:**
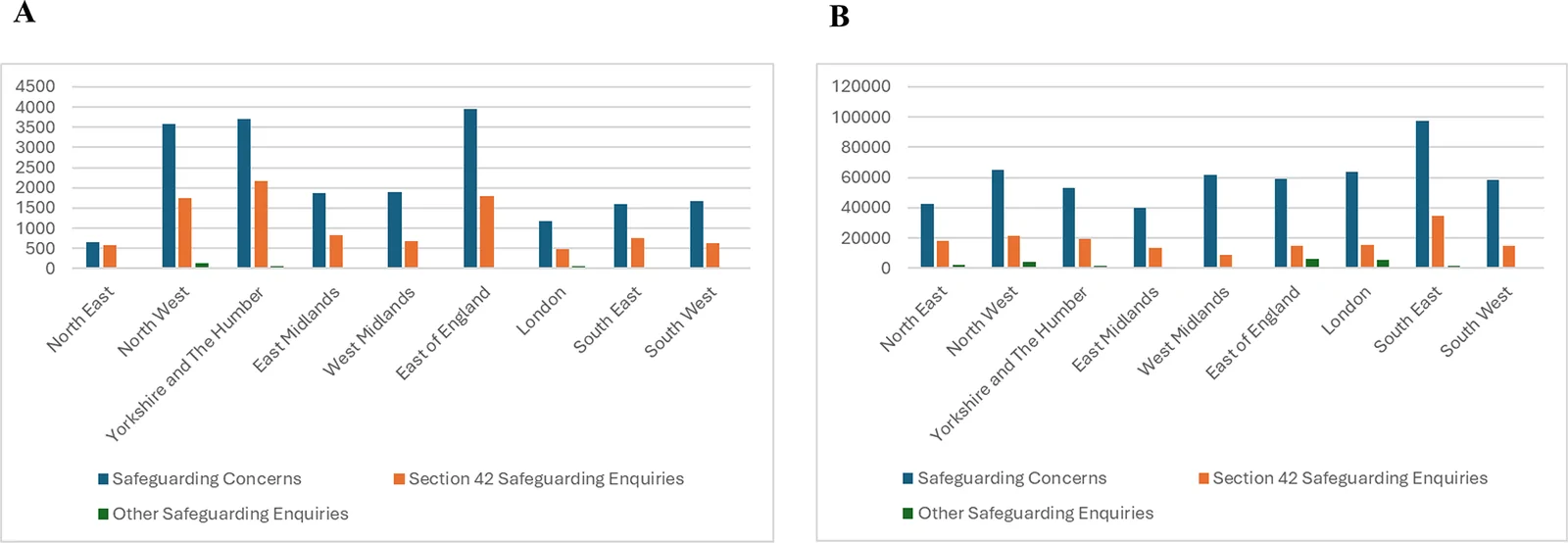
Counts of concerns and enquiries for (**A**) individuals with dementia and (**B**) all individuals by region 2021–2022. Source: National Health Service (NHS) Digital Safeguarding Adults, England.

Figure 2 presents the regional distribution of safeguarding concerns and enquiries for both (**A**) individuals with dementia and (**B**) all individuals in 2021 to 2022. It reveals substantial regional variations in safeguarding activity, highlighting key differences in reporting and intervention across England. For individuals with dementia (figure 2A), substantial regional variation in safeguarding concerns and enquiries was also observed. Among the regions, when safeguarding concerns and Section 42 enquiries were considered together, Yorkshire and the Humber recorded the highest combined number of cases (3705 safeguarding concerns and 2170 Section 42 enquiries). This was followed by the East of England (3955 safeguarding concerns, 1785 Section 42 enquiries) and the North West (3590 safeguarding concerns, 1735 Section 42 enquiries). In contrast, London (1175 concerns, 485 Section 42 enquiries), the South East (1600 concerns, 740 Section 42 enquiries), the South West (1675 concerns, 635 Section 42 enquiries) and the North East (660 concerns, 585 Section 42 enquiries) recorded the lowest levels of safeguarding activity for people with dementia.

Across all individuals (figure 2B), the South East recorded the highest total number of safeguarding concerns (97 730), followed by the North West (64 880) and London (63 755). For Section 42 Safeguarding enquiries, the South East (34 715), the North West (21 585) and Yorkshire and the Humber (19 685) reported the highest numbers. London (15 460) ranked below Yorkshire and the Humber, the North East (18 360), and the East of England (15 010) for Section 42 enquiries. East Midlands (40 210 concerns and 13 465 Section 42 Safeguarding enquiries) and West Midlands (61 970 concerns and 8960 Section 42 Safeguarding enquiries) had comparatively lower Section 42 enquiry numbers relative to their overall safeguarding concerns. Notably, while London recorded comparatively high safeguarding activity for the general population, it had one of the lowest figures for individuals with dementia, which may reflect differences in reporting or identification of safeguarding concerns in this group.

**Figure 3:**
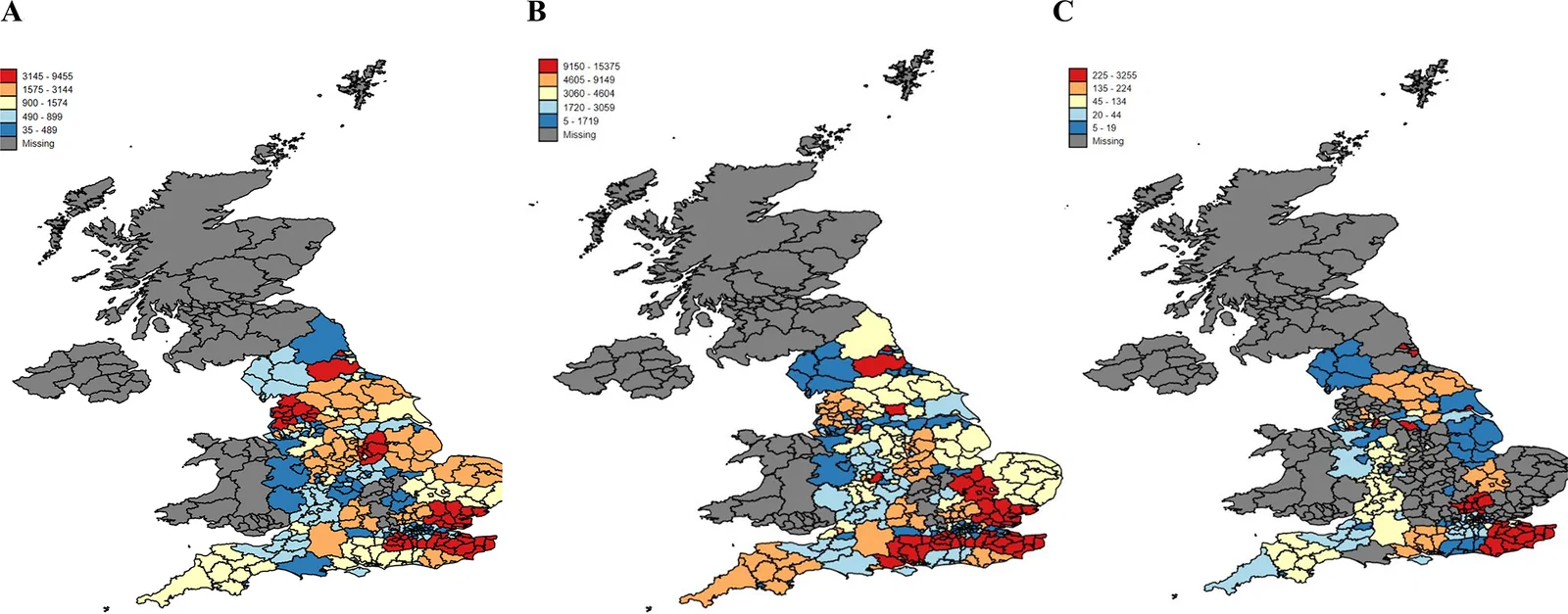
Spatial map of number of (**A**) safeguarding concerns; (**B**) Section 42 safeguarding enquiries and (**C**) other enquiries among all individuals (adults) in England (Source: National Health Service Digital 2021/2022). Maps present absolute counts of safeguarding activity, not adjusted for population size.

Figure 3 presents spatial maps illustrating the geographic distribution of safeguarding concerns (**A**), Section 42 Safeguarding Enquiries (**B**), and other enquiries (**C**) among all individuals (adults) across the 314 LADs in England for 2021 to 2022. The maps reveal substantial regional variation in safeguarding concerns and enquiries. Figure 3A shows that LADs in the highest safeguarding category were primarily concentrated in the South East, including Surrey, Hampshire, Buckinghamshire and Kent, as well as Essex in the East of England, Manchester in the North West, Birmingham in the West Midlands, Leeds in Yorkshire and the Humber and Newcastle-upon-Tyne and Durham in the North East. While London recorded a high regional total overall, no individual London borough fell within the highest category, reflecting the fact that London’s aggregate figure results from many mid-range boroughs rather than a small number of exceptionally high-recording districts. Many rural areas and districts in the North and Midlands recorded lower numbers of concerns, shown in the lighter colour bands. Figure 3B follows a similar pattern, with higher numbers of Section 42 safeguarding enquiries occurring in areas that also recorded higher levels of safeguarding concerns. However, some areas with high safeguarding concerns have fewer Section 42 enquiries, suggesting that not all reported concerns progress to a formal statutory intervention.

Figure 3C highlights a lower volume of Other safeguarding enquiries, with the highest numbers recorded in the South East, London and parts of the Midlands. These geographic patterns emphasise the need for targeted safeguarding interventions in high-risk areas and a standardised approach to reporting and intervention to ensure equitable protection for older adults and people living with dementia across England.

**Figure 4:**
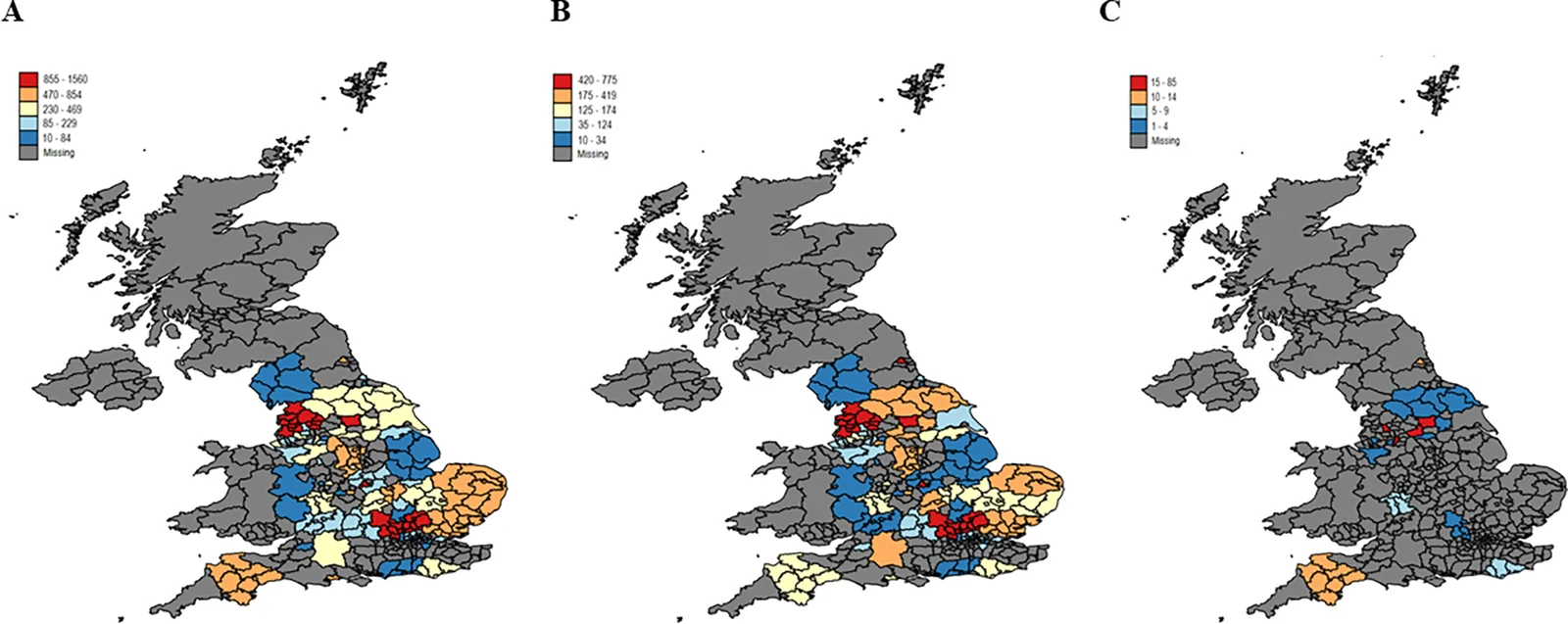
Spatial map of number of (**A**) safeguarding concerns; (**B**) Section 42 safeguarding enquiries and (**C**) other enquiries among individuals with dementia in England (Source: National Health Service Digital 2021/2022). Maps present absolute counts of safeguarding activity, not adjusted for population size.

Figure 4 presents spatial maps illustrating the regional distribution of safeguarding concerns (**A**), Section 42 Safeguarding Enquiries (**B**), and other safeguarding enquiries (**C**) specifically for individuals with dementia across England in 2021/2022. The maps indicate substantial regional variations in safeguarding reports, reflecting differences in dementia-related safeguarding risks, reporting rates and intervention practices across local authorities. Figure 4A shows that the highest numbers of safeguarding concerns among individuals with dementia (855–1560 cases) were recorded in a small number of individual LADs, including one district in the Midlands and several in the North West. Moderate levels of safeguarding concerns were observed across a wider set of local authorities represented by the intermediate colour bands in the figure, while many areas, particularly in the North East and more rural regions, recorded low numbers of concerns or had data suppressed due to disclosure control.

Figure 4B shows a broadly similar but not identical spatial pattern to figure 4A. While several LADs that recorded the highest safeguarding concerns also recorded the highest numbers of Section 42 enquiries, the relationship is mixed. Some districts move into a higher category in figure 4B relative to figure 4A, while others fall into a lower category, suggesting that the proportion of concerns progressing to formal Section 42 enquiries varies across local authorities. Figure 4C highlights that Other safeguarding enquiries for people with dementia are sparse across England, with the majority of LADs reporting fewer than 10 cases annually or having data suppressed due to disclosure control. Where higher numbers are visible, these appear in parts of the North West and Yorkshire and the Humber. Large areas across the North, East and rural regions have missing or suppressed data, reflecting the very low absolute numbers of Other safeguarding enquiries for this population.

## Discussion

This study makes an original contribution to the literature by identifying the temporal and geographical patterns of safeguarding among older people with dementia in England using three national datasets. This is significant, as there are no existing studies using Local Authority data which have examined safeguarding issues related to those living with dementia. Our study revealed substantial increases in safeguarding concerns and enquiries in England between 2015 and 2022, particularly concerning individuals with dementia. The overall rise in safeguarding concerns, which more than doubled during the study period, suggests an increasing recognition and reporting of safeguarding issues. This finding is consistent with a study of safeguarding data over different years, which indicates a trend of increasing inquiries.^23^ The increase in safeguarding concerns and enquiries among people living with dementia may represent increased awareness, guidance indicating the need to identify dementia abuse and neglect, and to address such abuse within the safeguarding system.^24 25^ In line with previous research,^23^ our figures suggest that increased levels of safeguarding correlate with areas of deprivation, where local councils face funding reductions amid rising needs for safeguarding services.^26^ However, higher activity may also reflect organisational differences in practice, such as variation in practitioner training, awareness and interagency collaboration across local authorities.^27 28^ This suggests that economic stability has a significant influence on the volume of safeguarding inquiries, potentially reflecting broader systemic inequalities.

Among individuals with dementia, similar regional patterns are observed, with higher safeguarding concerns reported in more deprived areas such as parts of the North West and Midlands. However, it is not clear from the data whether people with dementia experience disproportionately higher levels of safeguarding concerns compared with the general population. If higher levels are indeed present, this may reflect the fact that dementia often constitutes an evident care and support need under Section 42 of the Care Act 2014, making safeguarding concerns more likely to be raised. Notably, despite the rise in concerns, there is a relative dip in the number of cases that progress to formal enquiries, suggesting variability in how thresholds for Section 42 eligibility are applied. This may also indicate inconsistencies in local decision-making or resource availability that affect safeguarding pathways for this group.

However, the fluctuations in Section 42 Safeguarding Enquiries, particularly the decline in 2020/2021 in our study, indicate potential disruptions in safeguarding processes during the COVID-19 pandemic. Guidance to practitioners during the COVID-19 pandemic noted that people living with dementia were likely to be at higher risk of abuse and neglect than other populations, due to factors such as increased social isolation, caregiver burden and staffing pressures within residential homes.^29^ Despite these concerns, our findings show a decline in both safeguarding concerns and enquiries for individuals with dementia in 2020/2021. This suggests that the increased risks identified by Social Care Institute for Excellence may not have translated into increased safeguarding activity for this group, possibly reflecting challenges in detection and reporting.^29^ Reduced levels of safeguarding may reflect a reluctance among people with dementia and their carers to engage with services during the pandemic, as observed in previous research,^30^ alongside significant workforce shortages in the health and social care system.^31^ These findings raise important questions about the visibility and protection of people with dementia during health crises and underscore the need for safeguarding systems that remain responsive under emergency conditions.

The observed increase in concerns, combined with relatively stable numbers of Section 42 enquiries, suggests that while more cases are being reported, not all concerns escalate to formal safeguarding enquiries. In the case of people living with dementia, this may be because local authority workers have made a judgement that the Section 42 criteria are not met. While dementia may be identified as a care and support need under Section 42(a) of the Care Act 2014, the severity of dementia that a person may experience is highly variable. In other words, in some cases, it may be judged that a person’s dementia does not render them unable to protect themselves from abuse or neglect under Section 42(c) of the Care Act 2014. In addition to this, safeguarding research has noted differences in models and practices across local authorities.^30 31^ These differences may lead to different thresholds for intervention or resource availability across regions, affecting the degree to which people living with dementia with similar needs are judged to reach the threshold for Section 42 or for an ‘other’ enquiry or not.

Regional disparities in safeguarding activity may be affected by the unequal distribution of local authority resources across England, as highlighted in previous research.^26^ The regional disparities in safeguarding activity further emphasise the variation in reporting, intervention strategies and safeguarding risk factors across England. The highest safeguarding concerns and Section 42 enquiries were reported in the South East, North West and London, while regions such as the North East and parts of the Midlands had comparatively lower safeguarding activity. These differences may be influenced by population demographics, healthcare infrastructure and variations in local authority safeguarding policies. Additionally, while London recorded some of the highest safeguarding concerns for the general population, it had one of the lowest reported figures for individuals with dementia. This disparity may indicate under-reporting or lower identification of safeguarding risks, specifically among people with dementia. Possible explanations include lower dementia diagnosis rates, greater social isolation in urban settings or reduced engagement with health and social care services. It may also reflect variation in practitioner awareness of how dementia can present safeguarding risks^32^ or cultural and systemic barriers to reporting concerns within diverse communities in the capital.^33^

In terms of people living with dementia, research has shown that this group have higher levels of care needs and is of a lower socioeconomic status than those without dementia.^34^ Unmet care needs are concentrated among poorer individuals, both with and without dementia. As dementia places higher burdens on informal caregivers, a known risk factor for abuse and neglect,^2^ one would expect this to result in higher rates of safeguarding concerns and enquiries. In addition, methodological factors such as underdiagnosis in primary care registers, suppression of small numbers and London’s comparatively younger age profile may partly explain this anomaly. Taken together, these issues suggest that safeguarding activity for people with dementia may be underestimated in some regions, particularly in London.

### Strength and limitations of this study

This study presents a novel, data-driven analysis of safeguarding patterns among people living with dementia in England, leveraging national datasets routinely used for monitoring adult safeguarding and dementia prevalence. It is the first study, to our knowledge, to systematically explore the geographical and demographic distribution of safeguarding concerns and enquiries specifically for individuals with dementia, offering an important contribution to an under-researched area. By integrating data from NHS Digital, the ONS and dementia diagnosis records, the study offers a comprehensive overview of safeguarding activity across LADs. This approach enables a detailed examination of age, sex and regional trends in safeguarding, thereby generating evidence directly relevant to local policy planning and resource allocation. The use of spatial mapping further enhances the accessibility and utility of findings for policymakers, practitioners and researchers working to improve protection for older adults, including people living with dementia.

One limitation of this study is the low frequency of non-Section 42 safeguarding cases, with most areas reporting fewer than 10 cases annually. While the South East, London and parts of the Midlands recorded relatively higher numbers of other enquiries, many regions, particularly in the North and more rural areas, recorded very low numbers or had data suppressed due to disclosure control, limiting interpretation of geographic variation. This restriction poses a challenge in fully assessing safeguarding trends in low-reporting regions, potentially leading to an underestimation of safeguarding issues in these areas. Future research should explore alternative approaches to data aggregation and reporting to ensure a more comprehensive understanding of safeguarding patterns while maintaining privacy safeguards.

Another notable limitation of this study is the absence of disaggregated data on the type of abuse reported in safeguarding concerns or investigated under Section 42 enquiries. The national safeguarding dataset used does not provide information on whether concerns related to physical, financial, psychological, sexual or domestic abuse. This limits our ability to interpret the observed sex differences in safeguarding concerns, particularly the higher rates among older females. For example, if a significant proportion of concerns are related to domestic abuse, this might help explain the gender disparity, given that older women are more frequently affected by such abuse. The lack of an abuse-type breakdown restricts a more nuanced understanding of risk profiles and safeguarding needs, particularly for individuals with dementia. This data gap should be addressed at a policy level to support more targeted interventions and inform equitable safeguarding practices across demographic groups.

Research focusing on reports of elder abuse to telephone helplines indicates that people living with dementia are at high risk of experiencing multiple forms of abuse.^5^ However, the available safeguarding datasets are reported at the area level and do not include individual-level identifiers. As a result, it is not possible to determine whether multiple safeguarding concerns recorded in a local authority relate to separate individuals or to repeated reports about the same person. For example, a single individual may be the subject of multiple concerns or enquiries over time, but these would appear in the dataset as distinct cases. This limitation affects the ability to assess the true incidence of abuse and may lead to an overestimation of the number of individuals affected. Future research using linked, individual-level safeguarding data could examine the proportion of concerns that progress to enquiries over time and across regions, providing insight into variation in safeguarding thresholds, investigative capacity and local authority practice.

Another limitation of this study is the lack of publicly available data on safeguarding concerns and enquiries specifically disaggregated by age and sex for individuals with dementia. As a result, it was necessary to estimate the number of safeguarding cases by sex and age group in each region using NHS Digital Recorded Dementia Diagnoses 2022 and the 2021 ONS population projections for local authorities. While this approach enables a proportional distribution of safeguarding cases based on dementia prevalence and population estimates, it introduces potential uncertainties and assumptions that may impact the precision of our findings. In addition, safeguarding data are not disaggregated by ethnicity, which limits our ability to explore trends among minority ethnic groups, which is an important gap given evidence of inequalities in dementia diagnosis, service access and safeguarding experiences. Future studies would benefit from direct access to fully disaggregated safeguarding data, including by age, sex and ethnicity, to enable more accurate and detailed analyses of demographic risk factors. Finally, the geographical analyses were based on a single reporting year (2021/2022) and therefore provide a snapshot of safeguarding activity rather than a longitudinal assessment of spatial patterns. As a result, the observed geographic variation may not be fully representative of patterns across the entire 2015–2022 period.

### Policy implications

The findings of this study have important policy implications for safeguarding individuals with dementia in England. The substantial increase in safeguarding concerns over time highlights both the heightened safeguarding risks faced by older adults with cognitive impairments and the growing responsiveness of safeguarding systems. This rise may also reflect greater awareness, improved detection and more proactive reporting by professionals and the public. Policymakers should review and standardise safeguarding thresholds across local authorities to ensure consistency in decision-making and equitable access to protection services. Furthermore, policymakers have previously supported advocacy services as a means of protecting older adults from abuse and neglect.^35^ Greater advocacy services for people living with dementia could be commissioned to ensure that people living with dementia who are without family support or who are being abused by a family member are supported to register a safeguarding concern.

Among the general adult population, regions such as the South East and North West reported the highest safeguarding activity, both in terms of overall concerns and Section 42 enquiries, which may indicate greater underlying need but could also reflect differences in reporting practices and system responsiveness. Conversely, areas with lower reported concerns, such as the East Midlands and South West, may benefit from targeted awareness campaigns and support to strengthen detection and early intervention. For people living with dementia specifically, Yorkshire and the Humber, the East of England and the North West recorded the highest safeguarding activity, while London, the South East and the South West recorded the lowest, underscoring the importance of dementia-specific safeguarding strategies. Local authorities should be supported in enhancing their safeguarding frameworks, increasing workforce capacity and improving coordination between health and social care services to address safeguarding risks more effectively.

Lastly, there is a clear need for the Government to strengthen the quality and granularity of safeguarding data. The Safeguarding Adults Collection provides valuable information; however, its utility is limited as it is recorded only at the area level. Collecting data at the individual level would allow researchers to examine how factors such as dementia diagnosis, age, sex and ethnicity influence safeguarding activity. In addition, recording the reasons for Section 42 enquiries in a consistent typology and tracking how these reasons change over time would provide important insights into emerging risks, system responsiveness and the effectiveness of safeguarding policies.

## Supplementary material

10.1136/bmjopen-2025-110575 online supplemental table 1

## Data Availability

Data are available in a public, open access repository.
