# Supplementary material for: Geographical and temporal distribution of safeguarding concerns among older adults with dementia in England: a population-based repeated cross-sectional study
Source: BMJ Open. 2026 Aug 26;16(8):e110575. doi: 10.1136/bmjopen-2025-110575 (PMC13536066; doi:10.1136/bmjopen-2025-110575)
Supplement: Supplementary file 1 [file bmjopen-2025-110575.reviewer_comments.pdf]

PEER REVIEW HISTORY

BMJ Open publishes all reviews undertaken for accepted manuscripts. Reviewers are asked to complete a checklist review form and are provided with free text boxes to elaborate on their assessment. These free text comments are reproduced below.

ARTICLE DETAILS

Title (Provisional)

Geographical and temporal distribution of safeguarding concerns among older adults with dementia in England: a population-based repeated cross-sectional study

Authors

Maharani, Asri; Dixon, Jeremy; Szulewska, Katarzyna; Robinson, Catherine; Cooper, Mark; Panagioti, Maria; Blakeman, Thomas; Lasrado, Reena

---

VERSION 1 - REVIEW

**Reviewer**	**1**
**Name**	**Duffy, Anita**
**Affiliation**	**Our Lady's Hospice & Care Services, education**
**Date**	**28-Oct-2025**
**COI**	**None**

The fundamental concern I have regarding this paper is the lack of ethical approval to access, and report the data. If the editor is satisfied that the authors did not require ethical permission to undertake this research study then I recommend minor revision of the article.

Geographical and temporal distribution of safeguarding concerns among older adults with dementia in England: a population-based study_ Reviewer comments

The article provides a valuable and thought-provoking contribution to the literature on abuse and safeguarding among people living with dementia in England. The topic is of great importance, and offers significant insights that could enhance understanding and inform policy, research and practice in safeguarding care environments.

While the study's findings and discussions are creditable, there are some areas that require further clarification. In particular, minor issues could be refined to improve the paper. More importantly however, the justification the authors provide for not obtaining ethical approval raises concerns regarding the ethical rigour of the study warranting a clearer explanation. I suggest including a research question aims and objectives in the manuscript and clearly outline the research approach and methods used in the study.

Please see my comments below:

Page 3: Ethics statement: What UK regulations are the authors referring to regarding not needing ethical permission to access and publish the data. Most journal editors

look for ethical approval before accepting research studies for publication. The title of this paper and the authors identify this manuscript as a population-based study. Therefore, it is not clear why the authors did not apply for low risk ethical permission from the university REC to collect and publish the data.

Page 5 abstract (and page 9): can the authors explain why they only collected data up until 2022, especially for an article being peer reviewed in 2025 and also because NHS digital safeguarding adult's dataset is collected annually

Page 8: can the authors explain the range in gap in the statistics mentioned on line 123.

Page 8: Are the authors familiar with the work of Sarah Donnelly on carer harm (line 126). Her work may add to this section.

Page 8 what do the authors mean by 'fault lines'? (line 135)

Page 10: line 163 should 'data' be 'data set'?

Page 10 can the authors explain what they mean by other safeguarding enquiries (line 168)

Page 10: line 186 does other need a capital letter or is this a typographical error

Page 12 and page 22: please refer to the group as a people living with dementia rather than 'this vulnerable group', the term vulnerability is no longer an appropriate term when discussing groups at risk of abuse.

Page 22: please explain abbreviations before using them SCIE, some readers may not be familiar with this agency.

Page 31 & 32 (supplementary table 1) please do not refer to people living with dementia as 'dementia patients'

Reviewer 2

Name Bray, Jennifer

Affiliation University of Worcester, The Association for Dementia Studies, St Johns Campus

Date 08-Jan-2026

COI None

I thought this would be a really interesting article on an important topic, and while this was true to some extent it was let down by a lack of clarity and consistency in several places. It also felt as if there could be more attention to detail to ensure accuracy and avoid repetition. Hopefully the following comments will be seen as constructive rather than critical, as I really would like to see an improved version.

Line 97 – it should be 'this population is' not 'this population are'.

Line 104 – I was expecting to see a reference for the Care Act 2014 here when you first talk about it.

Line 123 – what do you mean by ‘reports of abuse ranging from 5.4% to 62.3%’? What is the % of? I’m it’s something to do with prevalence, but it’s not clear. Also, does it relate to abuse committed by family caregivers, or abuse reported by family caregivers, or maybe both? The wording is ambiguous.

Line 124 – it feels like ‘instances of abuse and neglect are particularly high’ would be better and clearer.

Line 128 – should it be ‘responses to people’ rather than ‘responses of people’?

Lines 157-160 – it currently is easy to read these lines as saying that in 2021-22 there were 541,535 safeguarding concerns and in 2022 451,992 people had a dementia diagnosis. I’m assuming that the figures actually relate to the period 2015-2022 as that’s the period you’re interested in according to line 145, but I might be wrong (I can see from later on that the 541,535 is just for 2021-22). If you actually only looked at data for one timepoint/year, I’m not sure how you used the data sources to analyse temporal trends, and I’m not clear why you are giving the numbers for one year here as they are not relevant to anything. Maybe the years are part of the name of the data source, causing unnecessary confusion, and you can actually access historical data via the source. Providing a bit of clarity would be useful. Also, do you actually need to give the numbers here regardless of what period they relate to, as that feels like the sort of thing that would come in the results section.

Lines 184-189 – I’m not sure what these sentences are supposed to add. Most of it has already been covered. The final sentence is new, but with no follow-up is not particularly helpful. If it’s to make the point that although enquiries might take a while, each enquiry is only recorded once in the year it was opened, then make this clear. Currently it just makes me wonder whether it’s actually recorded twice, once when it opens and once when it concludes, which I assume is not the case. Reworking this to provide clarity and avoid introducing uncertainty would be good.

Line 196 – you’ve already set up the ONS acronym in line 179 so you can just use it here.

Supplementary Table 1 – the first age range for England should be ‘0-29’ not ‘029’.

Line 199 – do you need to direct the reader to the supplementary table here? What does it add? I would wait until you are ready to talk about it in the results section (i.e. line 273), rather than encouraging the reader to look at it now and try to interpret it for themselves.

Line 201 – you’ve already set up the LADs acronym in line 153 so you can just use it here.

Line 204 – you’ve already set up the QOF acronym in line 175 so you can just use it here.

Results – a general point, but if enquiries result from concerns, would it be useful to say what % of concerns resulted in enquiries, as well as giving actual numbers? For example, if the concern-to-enquiry % reduces/increases over time that might be interesting to explore. E.g. could a low % suggest over-zealous reporting or an inability/unwillingness to investigate? Similarly, if one region has a particularly high or low % that could also be interesting, rather than it being overlooked because the actual numbers were average.

Lines 217-219 – I’m not sure this sentence is necessary, as you basically repeat it in lines 228-229. Could you rework it to just focus on Figure 1A?

Line 220 – I found this phrasing confusing at first. To make it clearer what you mean, and to tie in with how you present data in Figure 1A, it might be better to say ‘from 14,165 in 2015/2016 to a peak of 22,020 in 2019/2020’.

For consistency, throughout the article it would be better to use one notation in both the text and the figures, so decide whether you prefer 2021/2022 or 2021-22 and stick with it.

Lines 221-222 – the sentence ‘The dataset captured…with dementia.’ feels like it would make more sense at the start of the paragraph as it provides the overall context, and would also be better if it reiterated the period of interest. It currently breaks the flow of the paragraph as saying ‘However, a notable decline…’ makes more sense if it immediately follows the sentence ending ‘this vulnerable group.’

Line 225 – why is there “?

Lines 234-236 – this might just be me, so I’m not sure if you need to change anything or not, but when you say ‘a peak of 177,565 in 2019-20’ it makes me think that this was ‘the’ peak, so to then say that it was ‘184,515 in 2021-22’ (i.e. higher) was confusing. I would suggest that you could have 2021-22 as the peak but still say there was a decline in 2020-21 without needing to mention 2019-20. If you want to keep 2019-20 in there, don’t refer to it as ‘a peak’ but just that it rose to 177,565 in 2019-20, as it is potentially confusing to the reader.

Figure 2 – For consistency with Figure 1, I would present 2A as individuals with dementia, and 2B as all individuals, rather than having Figure 1 and Figure 2 presenting information the opposite way. Similarly, the text in lines 246-268 is annoying for the reader as it is in the opposite order (England, then dementia) to the text discussing Figure 1. Either start with the wider picture in both cases, then focus on dementia, or start with dementia and broaden out – don’t mix, be consistent. (having seen that the rest of the article seems to be the wider picture, then focusing on dementia, it might be easier to change Figure 1 and the accompanying text to match this order)

Figure 2 – both 2A and 2B have the final ‘total enquiries’ column cut off for the South West. However, I’m not sure why you have included the total enquiries as it doesn’t add anything and you didn’t include these numbers in Figure 1. Again, consistency would be good.

Lines 243-244 – why do you only look at the period 2021 to 2022? I know you don’t specify years in objective 2 (lines 149-151), but as it follows an objective where a time period of 2015-2022 was given, it would be easy for the reader to think that you would use the same data in each case. Also, lines 191-192 imply that you’re looking at the whole period, but actually when you refer to ‘temporal and geographical’ you mean you looked at them separately rather than together (e.g. geographical over time). If you only looked at 2021-2022, please make this clear from the start and throughout, and provide justification for doing so and why you chose this particular year period. Did you choose it at the start, or once you had already looked at other data? Was it the only period where you had all the information available? At present, the reader is left with lots of questions, and thinking that it would be good to know if 2021-2022 is representative of the situation since 2015 or an odd snapshot which shows a completely different picture to any other year.

Lines 252-255 – is there a full set of the number of concerns per 1,000 adults for each region? I'm not sure how much it adds necessarily, but by reporting the figures for a select few regions it makes the reader want to have the full picture for all regions. I'd suggest either including them all and saying what they show, or not including any (especially if they don't seem to show anything new/extra). Actually, would these figures be a fairer way to compare the regions, as the absolute number of concerns and enquiries doesn't take into account the wider context around number of people etc.?

Line 256 – I'm not sure how Figure 2B shows a 'similar regional pattern' when only one of the highest 3 regions in 2A is still in the highest 3 regions in 2B, and the lowest region in 2A is now comfortably mid-range. Did you mean something else by this phrase? E.g. more about 'substantial regional variations' rather than the positions of the individual regions? If so, you need to be clearer.

Line 257 – it's probably worth clarifying that Yorkshire and The Humber has the highest number when concerns and enquiries are combined, as based just on concerns it's the second highest region behind the East of England. As you discussed concerns and enquiries separately for Figure 2A, the reader may expect you to do the same for Figure 2B, so suddenly combining the two may be confusing.

Lines 259-262 – again you are providing extra information about a one region (concerns and enquiries per 1,000 people with dementia), but not for all, and with an extra level of detail (enquiries) than for all individuals. Although you give a bit more context about what this means, without the full picture it is almost meaningless. As before, I'd include the information for all regions and say what it shows (and be consistent with the information provided for all individuals), or not include any.

Lines 273-296 – it feels odd to present the data about sex and age here, as the previous bit was about geography, and the following section is also about geography, so this breaks the flow. If you discussed any age or sex differences/patterns/trends for the different regions I could understand it, but you only present the figures for England in the text. I would either expect to see some form or statement to say that there was nothing of interest noted for the different regions, or some discussion about age and sex in (at least) the regions of interest from Figure 2B.

Lines 284-286 – I'm not sure I agree with this sentence. Based on the values given in supplementary table 1, I think there are 148 concerns for males under 60 and 141 for females under 60, not 'fewer than 100 cases'.

Lines 289-290 – I'm not sure why you present the enquiry figures differently than the concerns. For the concerns you indicated which age groups had the highest number of concerns for each sex (which were different for males and females), but for enquiries you're only looking at the top male age groups and giving the respective female figures, rather than actually saying which female age groups had the highest figures. I would suggest being consistent, but I appreciate that you're trying to make the point in the next sentence about the gender gap widening in the older age groups. If you choose to stick with your current approach, you should say '1,548 enquiries for those two age groups respectively' to make it clearer.

Lines 298-300 – it's probably worth making it clear in the text that Figure 3 relates to all individuals, not just those with dementia. The same point as raised earlier also applies regarding why you only look at 2021-22.

Figures 3&4 – how did you decide the ranges for each colour code? The bands are not equal, so it feels a bit arbitrary. Making a few minor changes to the bands could easily result in a very different map, so giving the reader a bit of information about what was done would provide reassurance that you're presenting an accurate picture. It also feels like the bands should be exclusive (i.e. 5 - 1719, 1720 - 3060) rather than inclusive (i.e. 5 - 1720, 1720 - 3060). If a LAD had 1720 concerns, which colour band does it fall in?

Also, is the actual number of concerns/enquiries the best measure, or should you consider number per 1,000 people as previously to put the different LADs on an equal footing?

Lines 305-306 – I'm not sure I totally agree with this statement. By talking about the 'highest number of statutory safeguarding investigations' and 'most safeguarding concerns' it makes me look at the red parts of Figures 3A and 3B. Your sentence therefore implies that the regions that are red in Figure 3B should also be red in 3A but this is not the case in the middle of the country and North West area. Admittedly they are orange in 3A, but that should be 'higher levels of safeguarding concerns' not 'most safeguarding concerns'.

Line 306 – why do you use 'statutory safeguarding investigations' when you've not used this terminology previously, and Figure 3B does not use it either?

Line 309 & line 336 – similar to the previous point, why do you use different terminology here, saying 'non-Section 42 safeguarding cases' instead of 'other enquiries'? Some consistent throughout the text, and also with the figures, would be helpful.

Lies 322-327 – I'm not sure I'm looking at the same Figure 4A as I can't make the text match what is shown in the figure. You say that concerns were highest (i.e. red, as you refer to 855 to 1,550 – even though Figure 4A actually has red being 855 - 1560) in 'parts of the Midlands, the South East, and London' but as far as I can tell there is only one red LAD in the Midlands (not multiple 'parts'), and lots in the North West that you don't mention. You also refer to 'moderate levels' as '270-855 cases' and I can't work out if you're referring to the orange band and mistyped 470-855, or combined the orange and yellow and mistyped 230-855.

Lines 332-334 – I'm also struggling with Figure 4B as there's an almost direct match between the red LADs (i.e. high safeguarding concerns) in 4A and 4B, so unless you are also referring to orange LADs in 4A I'm not sure how the enquiries is lower. To avoid confusion, I think you need to be clearer about what you are referring to (e.g. is 'highest' just red and 'high' red plus orange?).

Lines 337-339 – again, I can't make the text and Figure 4C match. Based on the colours (which I'm not sure is actually enough to say anything about), I'd say that the 'higher numbers of other enquiries' were in the North West, Yorkshire & The Humber, and possibly the South West if you include the orange LADs, so I'm not sure why you say 'The South East, London, and selected regions in the Midlands' in the text. Please check and clarify.

Figure 4C – the dark blue band does not make sense. It says 5 - 5 so is that meaning that you're dark blue if you report exactly 5 other enquiries?

Discussion – I haven't actually checked to make sure that your findings are still accurate, as it feels like some may need updating once the previous comments are addressed. The same applies to your section on strengths and limitations, especially lines 447-448 where I'm not sure your claim is correct, and the policy implications section.

Line 382 – you haven't said what SCIE stands for.

Lines 390-401 (and possibly throughout) – is it 'Section 42' or 'section 42'? Please be consistent.

Lines 409-410 and lines 422-428 – please check for repetition as these both seem to talk about London in very similar ways, using almost identical language and phrasing in places.

Strengths and limitations of this study – assuming you add in your reasoning for just looking at the geographical aspects for 2021-22 rather than across the period 2015-2022, I would still expect to see it acknowledged in the limitations that your geographical findings only offer a snapshot of the situation which may/may not be representative of the whole period.

Line 480 – you've already said what ONS means, so you can just use it.

VERSION 1 - AUTHOR RESPONSE

Comments from 1st Reviewer #1

The fundamental concern I have regarding this paper is the lack of ethical approval to access, and report the data. If the editor is satisfied that the authors did not require ethical permission to undertake this research study then I recommend minor revision of the article.

Authors' response

Thank you for the comment. We confirm that formal ethical approval was not required for this study. The analysis was based exclusively on fully anonymised, aggregate-level secondary data obtained from publicly available national sources (NHS Digital Safeguarding Adults Collection, NHS Digital Recorded Dementia Diagnoses, and Office for National Statistics population projections). No individual-level data were accessed, and no identifiable personal information was used at any stage of the research.

In line with UK Health Research Authority guidance and NHS Digital governance requirements, studies using anonymised, aggregated secondary data for statistical analysis do not require research ethics committee approval. We have clarified this more explicitly in the Ethics Statement to ensure transparency and to address any potential ambiguity for readers and editors.

Comments from 2nd Reviewer #1

I thought this would be a really interesting article on an important topic, and while this was true to some extent it was let down by a lack of clarity and consistency in several places. It also felt as if there could be more attention to detail to ensure accuracy and avoid repetition. Hopefully the following comments will be seen as constructive rather than critical, as I really would like to see an improved version.

Line 97 – it should be 'this population is' not 'this population are'.

Authors' response

We thank the Reviewer for the detailed and constructive feedback. We have revised the phrase "this population are" to "this population is" in line 97.

Comments from 2nd Reviewer #2

Line 104 – I was expecting to see a reference for the Care Act 2014 here when you first talk about it.

Authors' response

We have included the citation and reference for the Care Act 2014:

UK Parliament. Care Act 2014. London; 2014. Available from: https://www.legislation.gov.uk/ukpga/2014/23/contents/enacted

Comments from 2nd Reviewer #3

Line 123 – what do you mean by 'reports of abuse ranging from 5.4% to 62.3%'? What is the % of? I'm it's something to do with prevalence, but it's not clear. Also, does it relate to abuse committed by family caregivers, or abuse reported by family caregivers, or maybe both? The wording is ambiguous.

Authors' response

We have revised the text to clarify that the reported percentages refer to the prevalence of abusive or neglectful behaviours self-reported by family caregivers of people living with dementia, and not to safeguarding-confirmed or administratively recorded abuse. We have also incorporated additional detail from Fang and Yan's review, noting that the majority of

included studies relied solely on family caregiver reports, to improve clarity and transparency.

"*A systematic review on 49 studies in Asia, Europe and the United States reported high prevalence of abusive behaviours self-reported by family caregivers of people living with dementia, with overall abuse ranging from 1.6% to 62.3%, psychological abuse from 9.7% to 62.3%, and physical abuse from 1.6% to 18.0%. These caregiver-reported prevalence estimates were generally higher than those reported in Western countries, where overall abuse ranged from 5.4% to 51.0%, physical abuse from 1.5% to 20.0%, and psychological abuse from 3.5% to 51.0%. The majority of the reviewed studies relied solely on reports from family caregivers.*"

Comments from 2nd Reviewer #4

Line 124 – it feels like 'instances of abuse and neglect are particularly high' would be better and clearer.

Authors' response

We have revised the sentence into: "Family caregiver studies have identified that instances of abuse and neglect are particularly high where carers are co-residing with the person, possibly due to stress and conflict brought about by the caregiving role."

Comments from 2nd Reviewer #5

Line 128 – should it be 'responses to people' rather than 'responses of people'?

Authors' response

We have revised the words into "response to people".

Comments from 2nd Reviewer #6

Lines 157-160 – it currently is easy to read these lines as saying that in 2021-22 there were 541,535 safeguarding concerns and in 2022 451,992 people had a dementia diagnosis. I'm assuming that the figures actually relate to the period 2015-2022 as that's the period you're interested in according to line 145, but I might be wrong (I can see from later on that the

541,535 is just for 2021-22). If you actually only looked at data for one timepoint/year, I'm not sure how you used the data sources to analyse temporal trends, and I'm not clear why you are giving the numbers for one year here as they are not relevant to anything. Maybe the years are part of the name of the data source, causing unnecessary confusion, and you can actually access historical data via the source. Providing a bit of clarity would be useful. Also, do you actually need to give the numbers here regardless of what period they relate to, as that feels like the sort of thing that would come in the results section.

Authors' response

We have revised the Methods section to clarify that safeguarding data from the NHS Digital Safeguarding Adults Collection were analysed annually over the period 2015–2022 to assess temporal trends, while the figures reported for 2021/2022 were presented only as an illustrative example of the most recent reporting year, not as the sole time point analysed.

We have also clarified that the NHS Digital Recorded Dementia Diagnoses dataset provides cross-sectional prevalence estimates (2022), which were used to inform proportional allocation by age and sex rather than to analyse temporal trends in dementia prevalence. To improve clarity and avoid confusion, we have removed unnecessary numerical counts from the data source description and ensured that all headline figures are now presented only in the Results section.

Comments from 2nd Reviewer #7

Lines 184-189 – I'm not sure what these sentences are supposed to add. Most of it has already been covered. The final sentence is new, but with no follow-up is not particularly helpful. If it's to make the point that although enquiries might take a while, each enquiry is only recorded once in the year it was opened, then make this clear. Currently it just makes me wonder whether it's actually recorded twice, once when it opens and once when it concludes, which I assume is not the case. Reworking this to provide clarity and avoid introducing uncertainty would be good.

Authors' response

We have revised this section to remove repetition and to clarify explicitly that each safeguarding enquiry is recorded once in the year it is opened, even if it concludes in a

subsequent reporting year. This clarification has been incorporated into the Methods section to improve transparency and avoid misinterpretation.

Comments from 2nd Reviewer #8

Line 196 – you've already set up the ONS acronym in line 179 so you can just use it here.

Authors' response

We have revised the manuscript and used only the acronym (ONS).

Comments from 2nd Reviewer #9

Supplementary Table 1 – the first age range for England should be '0-29' not '029'.

Authors' response

We have revised the text in Supplementary Table 1 into "0-29".

Comments from 2nd Reviewer #10

Line 199 – do you need to direct the reader to the supplementary table here? What does it add? I would wait until you are ready to talk about it in the results section (i.e. line 273), rather than encouraging the reader to look at it now and try to interpret it for themselves.

Authors' response

We have therefore removed the reference to the supplementary table from this section and now introduce it only in the Results section, where it is explicitly discussed.

Comments from 2nd Reviewer #11

Line 201 – you've already set up the LADs acronym in line 153 so you can just use it here.

Authors' response

We have revised the manuscript and used only the acronym (LADs).

Comments from 2nd Reviewer #12

Line 204 – you've already set up the QOF acronym in line 175 so you can just use it here.

Authors' response

We have revised the manuscript and used only the acronym (QOF).

Comments from 2nd Reviewer #13

Results – a general point, but if enquiries result from concerns, would it be useful to say what % of concerns resulted in enquiries, as well as giving actual numbers? For example, if the concern-to-enquiry % reduces/increases over time that might be interesting to explore. E.g. could a low % suggest over-zealous reporting or an inability/unwillingness to investigate? Similarly, if one region has a particularly high or low % that could also be interesting, rather than it being overlooked because the actual numbers were average.

Authors' response

Thank you for the comment. We agree that examining the proportion of safeguarding concerns that progress to enquiries, and how this varies over time or across regions, could provide valuable insights into safeguarding thresholds and practices. However, the Safeguarding Adults Collection reports concerns and enquiries as separate aggregate counts and does not allow linkage of individual concerns to subsequent enquiries, either within or across reporting years. As a result, it is not possible to calculate a valid concern-to-enquiry progression rate without risking over-interpretation of the administrative data.

To avoid drawing potentially misleading inferences, we have therefore focused on describing the temporal and geographic patterns separately in counts of concerns and enquiries. We acknowledge in the Discussion that variation in the relationship between concerns and enquiries may reflect differences in local authority thresholds, reporting practices, or resource availability, and we highlight this as an important area for future research should linked individual-level safeguarding data become available.

Comments from 2nd Reviewer #14

Lines 217-219 – I'm not sure this sentence is necessary, as you basically repeat it in lines 228-229. Could you rework it to just focus on Figure 1A?

Authors' response

We have revised this section to remove redundancy and reworked the earlier sentence to focus specifically on the trends shown in Figure 1A, improving clarity and flow.

Comments from 2nd Reviewer #15

Line 220 – I found this phrasing confusing at first. To make it clearer what you mean, and to tie in with how you present data in Figure 1A, it might be better to say 'from 14,165 in 2015/2016 to a peak of 22,020 in 2019/2020'.

Authors' response

We have revised the phrasing at line 220 to improve clarity and to align more closely with how the data are presented in Figure 1A, specifying the change from 14,165 in 2015/2016 to a peak of 22,020 in 2019/2020.

Comments from 2nd Reviewer #16

For consistency, throughout the article it would be better to use one notation in both the text and the figures, so decide whether you prefer 2021/2022 or 2021-22 and stick with it.

Authors' response

We have now standardised date notation throughout the manuscript and figures, using 2021/2022 consistently in both text and visuals to ensure clarity and consistency.

Comments from 2nd Reviewer #17

Lines 221-222 – the sentence 'The dataset captured…with dementia.' feels like it would make more sense at the start of the paragraph as it provides the overall context, and would also be better if it reiterated the period of interest. It currently breaks the flow of the paragraph as saying 'However, a notable decline…' makes more sense if it immediately follows the sentence ending 'this vulnerable group.'

Authors' response

We have restructured the paragraph to improve flow:

"Between 2015/2016 and 2021/2022, the dataset captured over 120,000 safeguarding concerns involving individuals living with dementia in England. Focusing on individuals with dementia (Figure 1A), safeguarding concerns increased by 55.45%, from 14,165 in

2015/2016 to a peak of 22,020 in 2019/2020, reflecting a gradual rise in reported safeguarding issues for this group. However, a notable decline occurred in 2020/2021, followed by a partial recovery in 2021/2022, although levels remained below the pre-pandemic peak."

<u>Comments from 2nd Reviewer #18</u>

Line 225 – why is there "?

<u>Authors' response</u>

We have removed the " in line 225.

<u>Comments from 2nd Reviewer #19</u>

Lines 234-236 – this might just be me, so I'm not sure if you need to change anything or not, but when you say 'a peak of 177,565 in 2019-20' it makes me think that this was 'the' peak, so to then say that it was '184,515 in 2021-22' (i.e. higher) was confusing. I would suggest that you could have 2021-22 as the peak but still say there was a decline in 2020-21 without needing to mention 2019-20. If you want to keep 2019-20 in there, don't refer to it as 'a peak' but just that it rose to 177,565 in 2019-20, as it is potentially confusing to the reader.

<u>Authors' response</u>

We have revised the wording to avoid ambiguity by describing the increase in 2019/2020 without labelling it as a peak, while retaining the description of the decline in 2020/2021 and the subsequent rise in 2021/2022.

"Section 42 safeguarding enquiries increased from 111,460 in 2015/2016 to 177,565 in 2019/2020, declined in 2020/2021, and then rose to 184,515 in 2021/2022."

<u>Comments from 2nd Reviewer #20</u>

Figure 2 – For consistency with Figure 1, I would present 2A as individuals with dementia, and 2B as all individuals, rather than having Figure 1 and Figure 2 presenting information the opposite way. Similarly, the text in lines 246-268 is annoying for the reader as it is in the opposite order (England, then dementia) to the text discussing Figure 1. Either start with the

wider picture in both cases, then focus on dementia, or start with dementia and broaden out – don't mix, be consistent. (having seen that the rest of the article seems to be the wider picture, then focusing on dementia, it might be easier to change Figure 1 and the accompanying text to match this order)

Authors' response

We have revised Figure 2 so that panel A presents findings for all individuals and panel B presents findings for individuals with dementia, aligning with the structure used elsewhere in the manuscript. We have also reordered the corresponding Results text (lines 246–268) to first describe the wider population before focusing on individuals with dementia, ensuring a consistent narrative throughout the paper.

Comments from 2nd Reviewer #21

Figure 2 – both 2A and 2B have the final 'total enquiries' column cut off for the South West. However, I'm not sure why you have included the total enquiries as it doesn't add anything and you didn't include these numbers in Figure 1. Again, consistency would be good.

Authors' response

Thank you for the comment. To address this, we have removed the total enquiries column from both panels of Figure 2 and corrected the figure layout so that all remaining columns are fully visible, including the South West.

Comments from 2nd Reviewer #22

Lines 243-244 – why do you only look at the period 2021 to 2022? I know you don't specify years in objective 2 (lines 149-151), but as it follows an objective where a time period of 2015-2022 was given, it would be easy for the reader to think that you would use the same data in each case. Also, lines 191-192 imply that you're looking at the whole period, but actually when you refer to 'temporal and geographical' you mean you looked at them separately rather than together (e.g. geographical over time). If you only looked at 2021-2022, please make this clear from the start and throughout, and provide justification for doing so and why you chose this particular year period. Did you choose it at the start, or once you had already looked at other data? Was it the only period where you had all the information available? At present, the reader is left with lots of questions, and thinking that it would be

good to know if 2021-2022 is representative of the situation since 2015 or an odd snapshot which shows a completely different picture to any other year.

Authors' response

We have now revised the manuscript to make explicit that temporal trends in safeguarding concerns and enquiries were examined for the period 2015–2022, whereas geographical analyses were conducted using data from a single reporting year (2021/2022). We have clarified from the outset, including in the Objectives and Methods sections, that temporal and geographical patterns were analysed separately rather than jointly. The year 2021/2022 was selected a priori for the geographical analyses because it was the most recent year with complete and comparable data across local authorities and aligned with the availability of dementia prevalence estimates used for proportional allocation. We have added a brief justification for this choice and acknowledged that the resulting spatial findings represent a snapshot that may not be fully representative of earlier years.

Comments from 2nd Reviewer #23

Lines 252-255 – is there a full set of the number of concerns per 1,000 adults for each region? I'm not sure how much it adds necessarily, but by reporting the figures for a select few regions it makes the reader want to have the full picture for all regions. I'd suggest either including them all and saying what they show, or not including any (especially if they don't seem to show anything new/extra). Actually, would these figures be a fairer way to compare the regions, as the absolute number of concerns and enquiries doesn't take into account the wider context around number of people etc.?

Authors' response

We thank the reviewer for this thoughtful suggestion. We agree that presenting safeguarding activity relative to population size can facilitate fairer regional comparison. However, as this study focuses primarily on describing patterns of safeguarding activity using routinely reported administrative counts, we chose to present absolute numbers consistently across figures and text to maintain comparability with national safeguarding reports.

To avoid selective reporting and potential confusion, we have revised the Results section to remove references to per-1,000 population figures for selected regions and now focus consistently on absolute counts. We note in the Discussion that population-standardised rates

may offer additional insight and represent an important area for future research, particularly if applied systematically across all regions.

<u>Comments from 2nd Reviewer #24</u>

Line 256 – I'm not sure how Figure 2B shows a 'similar regional pattern' when only one of the highest 3 regions in 2A is still in the highest 3 regions in 2B, and the lowest region in 2A is now comfortably mid-range. Did you mean something else by this phrase? E.g. more about 'substantial regional variations' rather than the positions of the individual regions? If so, you need to be clearer.

<u>Authors' response</u>

We have revised the wording "similar regional pattern" to make clear that the key similarity relates to the presence of substantial regional variation, rather than the specific positions of individual regions.

"For individuals with dementia (Figure 2B), substantial regional variation in safeguarding concerns and enquiries was also observed."

<u>Comments from 2nd Reviewer #25</u>

Line 257 – it's probably worth clarifying that Yorkshire and The Humber has the highest number when concerns and enquiries are combined, as based just on concerns it's the second highest region behind the East of England. As you discussed concerns and enquiries separately for Figure 2A, the reader may expect you to do the same for Figure 2B, so suddenly combining the two may be confusing.

<u>Authors' response</u>

We have revised the text to clarify that Yorkshire and the Humber had the highest number when concerns and enquiries were considered together, while the East of England had the highest number of safeguarding concerns alone. This revision improves consistency with the presentation used for Figure 2A.

"Among the regions, when safeguarding concerns and Section 42 enquiries were considered together, Yorkshire and the Humber recorded the highest combined number of cases (3,705 safeguarding concerns and 2,170 Section 42 enquiries)."

Comments from 2nd Reviewer #26

Lines 259-262 – again you are providing extra information about a one region (concerns and enquiries per 1,000 people with dementia), but not for all, and with an extra level of detail (enquiries) than for all individuals. Although you give a bit more context about what this means, without the full picture it is almost meaningless. As before, I'd include the information for all regions and say what it shows (and be consistent with the information provided for all individuals), or not include any.

Authors' response

We have removed the region-specific per-1,000 figures from this section and now present findings consistently using absolute counts across regions.

Comments from 2nd Reviewer #27

Lines 273-296 – it feels odd to present the data about sex and age here, as the previous bit was about geography, and the following section is also about geography, so this breaks the flow. If you discussed any age or sex differences/patterns/trends for the different regions I could understand it, but you only present the figures for England in the text. I would either expect to see some form or statement to say that there was nothing of interest noted for the different regions, or some discussion about age and sex in (at least) the regions of interest from Figure 2B.

Authors' response

We have revised the structure of the Results section so that age- and sex-specific findings are presented as a distinct subsection describing national demographic patterns, positioned before the regional analyses.

Comments from 2nd Reviewer #28

Lines 284-286 – I'm not sure I agree with this sentence. Based on the values given in supplementary table 1, I think there are 148 concerns for males under 60 and 141 for females under 60, not 'fewer than 100 cases'.

Authors' response

We have revised the sentence to reflect the correct values shown in Supplementary Table 1, indicating that safeguarding concerns among individuals under 60 were low but exceeded 100 cases for both males and females.

"Safeguarding concerns among individuals under 60 were relatively low compared with older age groups, with approximately 148 cases among males and 141 cases among females."

Comments from 2nd Reviewer #29

Lines 289-290 – I'm not sure why you present the enquiry figures differently than the concerns. For the concerns you indicated which age groups had the highest number of concerns for each sex (which were different for males and females), but for enquiries you're only looking at the top male age groups and giving the respective female figures, rather than actually saying which female age groups had the highest figures. I would suggest being consistent, but I appreciate that you're trying to make the point in the next sentence about the gender gap widening in the older age groups. If you choose to stick with your current approach, you should say '1,548 enquiries for those two age groups respectively' to make it clearer.

Authors' response

We have revised the wording to make explicit that the female figures refer to the same age groups as those reported for males.

"The number of Section 42 Safeguarding Enquiries was estimated to follow a similar trend, with the highest number of enquiries occurring in the 80-84 (866 enquiries) and 85-89 (819 enquiries) age groups for males. While for females, the corresponding figures for these two age groups were estimated to be significantly higher, at 1,326 and 1,548 enquiries, respectively."

Comments from 2nd Reviewer #30

Lines 298-300 – it's probably worth making it clear in the text that Figure 3 relates to all individuals, not just those with dementia. The same point as raised earlier also applies regarding why you only look at 2021-22.

Authors' response

We have revised the text to clearly state this at first mention. We have also reiterated that the spatial analyses are based on data from the most recent reporting year (2021/2022), and clarified this consistently across the Results and Methods sections, with justification provided for this choice.

Comments from 2nd Reviewer #31

Figures 3&4 – how did you decide the ranges for each colour code? The bands are not equal, so it feels a bit arbitrary. Making a few minor changes to the bands could easily result in a very different map, so giving the reader a bit of information about what was done would provide reassurance that you're presenting an accurate picture. It also feels like the bands should be exclusive (i.e. 5 - 1719, 1720 - 3060) rather than inclusive (i.e. 5 - 1720, 1720 - 3060). If a LAD had 1720 concerns, which colour band does it fall in?

Also, is the actual number of concerns/enquiries the best measure, or should you consider number per 1,000 people as previously to put the different LADs on an equal footing?

Authors' response

We have now clarified how the colour bands were defined and ensured that category ranges are mutually exclusive to avoid ambiguity. Specifically, ranges are now defined using non-overlapping intervals (e.g. 5–1719, 1720–3060), and this is stated explicitly in the figure captions.

The colour bands were derived using data-driven groupings to reflect the highly skewed distribution of safeguarding counts across local authority districts, while maintaining interpretability given disclosure-controlled small numbers. We have added a brief explanation of this approach to the figure captions.

With respect to the use of absolute counts rather than population-standardised rates, we agree that rates per population can facilitate comparison across areas. However, to maintain consistency with national safeguarding reporting practices and to avoid introducing selective or partial rate-based comparisons, we have retained absolute counts throughout the figures and text. We now note this explicitly and highlight population-standardised mapping as an important area for future research where denominators can be applied consistently across all local authorities.

Comments from 2nd Reviewer #32

Lines 305-306 – I'm not sure I totally agree with this statement. By talking about the 'highest number of statutory safeguarding investigations' and 'most safeguarding concerns' it makes me look at the red parts of Figures 3A and 3B. Your sentence therefore implies that the regions that are red in Figure 3B should also be red in 3A but this is not the case in the middle of the country and North West area. Admittedly they are orange in 3A, but that should be 'higher levels of safeguarding concerns' not 'most safeguarding concerns'.

Authors' response

We have revised the text to describe higher levels of safeguarding concerns rather than the highest levels, thereby avoiding any implication of exact overlap between Figures 3A and 3B.

Comments from 2nd Reviewer #33

Line 306 – why do you use 'statutory safeguarding investigations' when you've not used this terminology previously, and Figure 3B does not use it either?

Authors' response

We have removed the wording 'statutory safeguarding investigations' and revised the text to use "Section 42 safeguarding enquiries" consistently throughout.

Comments from 2nd Reviewer #34

Line 309 & line 336 – similar to the previous point, why do you use different terminology here, saying 'non-Section 42 safeguarding cases' instead of 'other enquiries'? Some consistent throughout the text, and also with the figures, would be helpful.

Authors' response

We have revised the text at lines 309 and 336 to use the term "Other safeguarding enquiries" consistently throughout the manuscript, in line with the terminology used in the figures and elsewhere in the text.

Comments from 2nd Reviewer #35

Lies 322-327 – I'm not sure I'm looking at the same Figure 4A as I can't make the text match what is shown in the figure. You say that concerns were highest (i.e. red, as you refer to 855 to 1,550 – even though Figure 4A actually has red being 855 - 1560) in 'parts of the Midlands, the South East, and London' but as far as I can tell there is only one red LAD in the Midlands (not multiple 'parts'), and lots in the North West that you don't mention. You also refer to 'moderate levels' as '270-855 cases' and I can't work out if you're referring to the orange band and mistyped 470-855, or combined the orange and yellow and mistyped 230-855.

Authors' response

We have revised the description to align precisely with the colour bands used in the figure, corrected the numerical ranges, and avoided overstating the geographic concentration of the highest category. The revised text now refers to individual local authority districts rather than "parts" of regions and accurately reflects the distribution shown in the map.

"Figure 4A shows that the highest numbers of safeguarding concerns among individuals with dementia (855–1,560 cases) were recorded in a small number of individual local authority districts, including one district in the Midlands and several in the North West. Moderate levels of safeguarding concerns were observed across a wider set of local authorities represented by the intermediate colour bands in the figure, while many areas, particularly in the North East and more rural regions, recorded low numbers of concerns or had data suppressed due to disclosure control."

Comments from 2nd Reviewer #36

Lines 332-334 – I'm also struggling with Figure 4B as there's an almost direct match between the red LADs (i.e. high safeguarding concerns) in 4A and 4B, so unless you are also referring to orange LADs in 4A I'm not sure how the enquiries is lower. To avoid confusion, I think you need to be clearer about what you are referring to (e.g. is 'highest' just red and 'high' red plus orange?).

Authors' response

We have revised the text to make explicit that references to "highest" safeguarding concerns or enquiries correspond to the highest colour category only, and that comparisons with "high" or "moderate" levels refer to combined upper categories where relevant. The revised wording

now avoids implying discrepancies between Figures 4A and 4B where similar colour patterns are observed.

<u>Comments from 2nd Reviewer #37</u>

Lines 337-339 – again, I can't make the text and Figure 4C match. Based on the colours (which I'm not sure is actually enough to say anything about), I'd say that the 'higher numbers of other enquiries' were in the North West, Yorkshire & The Humber, and possibly the South West if you include the orange LADs, so I'm not sure why you say 'The South East, London, and selected regions in the Midlands' in the text. Please check and clarify.

<u>Authors' response</u>

We have rechecked the figure and underlying data and agree that the original wording did not accurately reflect the spatial distribution shown. We have revised the text to align more closely with Figure 4C and to avoid over-interpretation given the small numbers and disclosure-controlled categories used for other enquiries.

<u>Comments from 2nd Reviewer #38</u>

Figure 4C – the dark blue band does not make sense. It says 5 - 5 so is that meaning that you're dark blue if you report exactly 5 other enquiries?

<u>Authors' response</u>

We have revised the figure legend and classification of the dark blue band to "1-5" to ensure that category ranges are clearly defined and interpretable, and that no single-value bands are presented in a way that could cause ambiguity.

<u>Comments from 2nd Reviewer #39</u>

Discussion – I haven't actually checked to make sure that your findings are still accurate, as it feels like some may need updating once the previous comments are addressed. The same applies to your section on strengths and limitations, especially lines 447-448 where I'm not sure your claim is correct, and the policy implications section.

<u>Authors' response</u>

Thank you for the comment. Following the revisions made in response to the earlier comments, particularly those relating to clarification of data sources, temporal coverage, and geographical analyses, we have carefully reviewed and updated the Discussion, Strengths and limitations, and Policy Implications sections to ensure that all interpretations and claims remain accurate and appropriately supported by the revised analyses. Where necessary, wording has been refined to improve precision and to ensure consistency with the updated Results.

Comments from 2nd Reviewer #40

Line 382 – you haven't said what SCIE stands for.

Authors' response

We have expanded the abbreviation at first mention to Social Care Institute for Excellence (SCIE).

Comments from 2nd Reviewer #41

Lines 390-401 (and possibly throughout) – is it 'Section 42' or 'section 42'? Please be consistent.

Authors' response

We have revised the word into "Section 42" throughout document.

Comments from 2nd Reviewer #42

Lines 409-410 and lines 422-428 – please check for repetition as these both seem to talk about London in very similar ways, using almost identical language and phrasing in places.

Authors' response

We have revised the Discussion to remove duplicated language in lines 422-428.

Comments from 2nd Reviewer #43

> Strengths and limitations of this study – assuming you add in your reasoning for just looking at the geographical aspects for 2021-22 rather than across the period

2015-2022, I would still expect to see it acknowledged in the limitations that your geographical findings only offer a snapshot of the situation which may/may not be representative of the whole period.

Authors' response

We agree that the geographical analyses, which focus on the most recent reporting year (2021/2022), provide a snapshot rather than a longitudinal assessment of spatial patterns. We have now explicitly acknowledged this in the Strengths and limitations of this study section, noting that the geographical findings may not be fully representative of patterns across the entire 2015–2022 period.

Comments from 2nd Reviewer #44

Line 480 – you've already said what ONS means, so you can just use it.

Authors' response

We have revised the manuscript and used only the acronym (ONS).

VERSION 2 - REVIEW

Reviewer 2

Name Bray, Jennifer

Affiliation University of Worcester, The Association for Dementia Studies, St Johns Campus

Date 26-Mar-2026

COI

Thank you for taking on board the previous comments. Unfortunately, some changes have introduced additional issues, while I'm nor sure that all comments have actually been addressed. It's a bit of a shame as I previously raised that attention to detail was an issue, and that still seems to be the case.

Line 70 – I appreciate you reference the Care Act in line 82, but it would make sense to include the reference here as well, when you first talk about the Care Act.

Line 71 – it should be 'apply' not 'appliy'

Lines 123-124 – should this be a bullet point as it doesn't really work as a standalone sentence? Also, the 'Approach' heading appears to have ended up at the end of the sentence rather than on a separate line.

In Line 131 you say 'Office for National Statistics 2021' but in line 149 you say 'The 2020 Office for National Statistics', then in line 163 it's 'the 2021 ONS'. Which year is correct?

Line 157 – the 'Data Analysis' sub-heading appears to have ended up at the end of the sentence rather than on a separate line.

Line 169 – you use 'LAD' but haven't said what it stands for (the bit where you explained it has since been deleted). Also, you say '317 LADs' but later on in line 263 you say '314 LADs', so which is correct?

Line 222 – the word 'approximately' is not needed, as you've given the actual values, not approximations.

Lines 242-243 – saying 'Despite the high safeguarding concerns and Section 42 enquiries for general population' doesn't really make sense at this point as you haven't looked at the general population yet (the order of Figures 2A and 2B changed in this version). It also seems odd not to mention the North East which has the lowest numbers, but is currently not included. It might make more sense to talk about the three regions with the lowest numbers instead. If you want to make the point about regions having different patterns for people with dementia compared to the general population, do this when talking about Figure 2B.

Lines 249-250 – you are saying here that London is one of the three regions with the highest Section 42 Safeguarding enquiries, but from Figure 2B I would argue that after the South East and North West, both Yorkshire and the Humber and the North East should come before London.

Lines 288-289 – is there a reason why the red local authority districts in the South East/London regions are not mentioned?

Lines 296-297 – I'm not sure I agree with this sentence as several districts that were pale yellow in Figure 4A are now orange in Figure 4B, but can see that several other districts have done the opposite. Not sure if it needs clarifying or not.

Lines 299-301 – unless the image used in Figure 4C has changed (it's difficult to know, as it looks like it's been deleted completely to me), I'm not sure that it matches the text. I raised this in my previous review comments (#37 in the response to review comments document) and you said that you agreed and had revised the text, but from what I can see no track changes have been made to this text at all. My previous point therefore still stands that your text does not accurately reflect Figure 4C.

Lines 367-370 – it would be worthwhile clarifying that you are referring to the general population here. Also, as per an earlier comment I'm not convinced London fits with what

you say in terms of Section 42 enquiries. I appreciate it's tricky to have too much nuance and detail when providing a summary, but it does feel slightly misleading.

Figures – you say in your response to review comments that you have made changes to the Figures (e.g. category ranges), but I cannot see all of these in the version I have. E.g. Figure 4C still has the dark blue band as having the range '5-5', and Figures 4A, 4B and 4C all still have overlapping intervals such as '10-35', '35-125'.

VERSION 2 - AUTHOR RESPONSE

Comments from 2nd Reviewer #1:

Reviewer comment: Thank you for taking on board the previous comments. Unfortunately, some changes have introduced additional issues, while I'm nor sure that all comments have actually been addressed. It's a bit of a shame as I previously raised that attention to detail was an issue, and that still seems to be the case.

Line 70 - I appreciate you reference the Care Act in line 82, but it would make sense to include the reference here as well, when you first talk about the Care Act.

Author's Response:

Thank you for this suggestion. We have added a citation to the Care Act 2014 at line 70, at first mention, to ensure the reference is provided at the point of introduction.

Comments from 2nd Reviewer #2:

Reviewer comment: Line 71 - it should be 'apply' not 'appliy'

Author's Response:

We have corrected the typographical error at line 71 from "appliy" to "apply".

Comments from 2nd Reviewer #3:

Reviewer comment: Lines 123-124 - should this be a bullet point as it doesn't really work as a standalone sentence? Also, the 'Approach' heading appears to have ended up at the end of the sentence rather than on a separate line.

Author's Response:

Thank you for identifying these formatting issues. We have added the missing bullet point to the third objective at lines 123–124 so that it is consistent with the two preceding objectives, and we have ensured the 'Approach' heading sits on a separate line with a clear paragraph break before it.

Comments from 2nd Reviewer #4:

Reviewer comment: In line 131 you say 'Office for National Statistics 2021' but in line 149 you say 'The 2020 Office for National Statistics', then in line 163 it's 'the 2021 ONS'. Which year is correct?

Author's Response:

The correct year is 2021, referring to the ONS 2021 Population Projections for Local Authorities. We have reviewed the manuscript and ensured all references now consistently read "the 2021 ONS Population Projections for Local Authorities" throughout, including in the Data Sources section, the Data Analysis section, and the Limitations section.

Comments from 2nd Reviewer #5:

Reviewer comment: Line 157 - the 'Data Analysis' sub-heading appears to have ended up at the end of the sentence rather than on a separate line.

Author's Response:

We have moved the 'Data Analysis' sub-heading to its own separate line, consistent with the formatting of other sub-headings in the Methods section.

Comments from 2nd Reviewer #6:

Reviewer comment: Line 169 - you use 'LAD' but haven't said what it stands for (the bit where you explained it has since been deleted). Also, you say '317 LADs' but later on in line 263 you say '314 LADs', so which is correct?

Author's Response:

We have re-introduced the full definition at first use, so the text now reads 'Local Authority Districts (LADs)' at line 169. We have corrected '317' to '314' at line 169 and verified consistency throughout the manuscript.

Comments from 2nd Reviewer #7:

Reviewer comment: Line 222 - the word 'approximately' is not needed, as you've given the actual values, not approximations.

Author's Response:

We have omitted the word "approximately" in line 222.

Comments from 2nd Reviewer #8:

Reviewer comment: Lines 242 - saying 'Despite the high safeguarding concerns and Section 42 enquiries for general population' doesn't really make sense at this point as you haven't looked at the general population yet (the order of Figures 2A and 2B changed in this version). It also seems odd not to mention the North East which has the lowest numbers, but is currently not included. It might make more sense to talk about the three regions with the lowest numbers instead. If you want to

make the point about regions having different patterns for people with dementia compared to the general population, do this when talking about Figure 2B.

Author's Response:

Thank you for the comments. Addressing these, we have restructured the paragraph. The sentence referring to the general population has been removed from the discussion of Figure 2A, and the North East has been added as one of the three regions with the lowest safeguarding activity for people with dementia. Any comparison with the general population is made only in the Figure 2B paragraph.

Figure 2 presents the regional distribution of safeguarding concerns and enquiries for both (A) individuals with dementia and (B) all individuals in 2021 to 2022. It reveals substantial regional variations in safeguarding activity, highlighting key differences in reporting and intervention across England. For individuals with dementia (Figure 2A), substantial regional variation in safeguarding concerns and enquiries was also observed. Among the regions, when safeguarding concerns and Section 42 enquiries were considered together, Yorkshire and the Humber recorded the highest combined number of cases (3,705 safeguarding concerns and 2,170 Section 42 enquiries). This was followed by the East of England (3,955 safeguarding concerns, 1,785 Section 42 enquiries) and the North West (3,590 safeguarding concerns, 1,735 Section 42 enquiries). In contrast, London (1,175 concerns, 485 Section 42 enquiries), the South East (1,600 concerns, 740 Section 42 enquiries), the South West (1,675 concerns, 635 Section 42 enquiries), and the North East (660 concerns, 585 Section 42 enquiries) recorded the lowest levels of safeguarding activity for people with dementia.

Across all individuals (Figure 2B), the South East recorded the highest total number of safeguarding concerns (97,730), followed by the North West (64,880) and London (63,755). For Section 42 Safeguarding enquiries, the South East (34,715), the North West (21,585), and Yorkshire and the Humber (19,685) reported the highest numbers. London (15,460) ranked below Yorkshire and the Humber, the North East (18,360), and the East of England (15,010) for Section 42 enquiries. East Midlands (40,210 concerns and 13,465 Section 42 Safeguarding enquiries) and West Midlands (61,970 concerns and 8,960 Section 42 Safeguarding enquiries) had comparatively lower Section 42 enquiry numbers relative to their overall safeguarding concerns. Notably, while London recorded comparatively high safeguarding activity for the general population, it had one of the lowest figures for individuals with dementia, which may reflect differences in reporting or identification of safeguarding concerns in this group.

Comments from 2nd Reviewer #9:

Reviewer comment: Lines 249-250 - you are saying here that London is one of the three regions with the highest Section 42 Safeguarding enquiries, but from Figure 2B, Yorkshire and the Humber and the North East should come before London.

Author's Response:

Having re-examined the data, we confirm that for Section 42 Safeguarding enquiries among the general population, the three highest regions are the South East (34,715), the North West (21,585), and Yorkshire and the Humber (19,685). London (15,460) in fact ranks below not only Yorkshire and the Humber but also the North East (18,360) and the East of England (15,010). We have corrected the text accordingly and London is now discussed separately in the context of the contrast between

its comparatively high overall safeguarding activity for the general population and its notably low figures for individuals with dementia.

Comments from 2nd Reviewer #10:

Reviewer comment: Lines 288-289 - is there a reason why the red local authority districts in the South East/London regions are not mentioned?

Author's Response:

We thank the Reviewer for this observation. We have revised the text to explicitly acknowledge the red local authority districts visible in Figure 3A. These are primarily concentrated in the South East (including Surrey, Hampshire, Buckinghamshire, and Kent), Essex in the East of England, Manchester in the North West, Birmingham in the West Midlands, Leeds in Yorkshire and the Humber, and Newcastle-upon-Tyne and Durham in the North East. Notably, while London recorded a high regional total overall, no individual London borough fell within the highest safeguarding category, reflecting the fact that London's aggregate figure results from many mid-range boroughs rather than a small number of exceptionally high-recording districts. We have revised the text to reflect this distinction.

Comments from 2nd Reviewer #11:

Reviewer comment: Lines 296-297 I'm not sure I agree with this sentence as several districts that were pale yellow in Figure 4A are now orange in Figure 4B, but can see that several other districts have done the opposite. Not sure if it needs clarifying or not.

Author's Response:

We agree that the relationship between Figures 4A and 4B is not straightforward — while some districts that recorded high safeguarding concerns also recorded high Section 42 enquiries, others show a different pattern, with some districts moving to a higher category in Figure 4B relative to 4A, and others to a lower one. We have revised the text to describe this as a mixed pattern and to avoid implying a direct correspondence between the two maps.

Figure 4B shows a broadly similar but not identical spatial pattern to Figure 4A. While several local authority districts that recorded the highest safeguarding concerns also recorded the highest numbers of Section 42 enquiries, the relationship is mixed. Some districts move into a higher category in Figure 4B relative to Figure 4A, while others fall into a lower category, suggesting that the proportion of concerns progressing to formal Section 42 enquiries varies across local authorities.

Comments from 2nd Reviewer #12:

Reviewer comment: Lines 299-301 - unless the image used in Figure 4C has changed (it's difficult to know, as it looks like it's been deleted completely to me), I'm not sure that it matches the text. I raised this in my previous review comments (#37 in the response to review comments document) and you said that you agreed and had revised the text, but from what I can see no track changes have been made to this text at all. My previous point therefore still stands that your text does not accurately reflect Figure 4C

Author's Response:

We have now carefully reviewed Figure 4C and revised the text accordingly. As the reviewer correctly noted in the previous round, the areas showing relatively higher numbers of Other safeguarding enquiries for people with dementia appear in parts of the North West and Yorkshire and the Humber, rather than the South East, London, and Midlands as previously stated. We have corrected the text and ensured track changes are clearly visible in the marked copy submitted with this revision.

Figure 4C highlights that Other safeguarding enquiries for people with dementia are sparse across England, with the majority of local authority districts reporting fewer than 10 cases annually or having data suppressed due to disclosure control. Where higher numbers are visible, these appear in parts of the North West and Yorkshire and the Humber. Large areas across the North, East, and rural regions have missing or suppressed data, reflecting the very low absolute numbers of Other safeguarding enquiries for this population.

<u>Comments from 2nd Reviewer #13:</u>

Reviewer comment: Lines 367-370 - it would be worthwhile clarifying that you are referring to the general population here. Also, as per an earlier comment I'm not convinced London fits with what you say in terms of Section 42 enquiries. I appreciate it's tricky to have too much nuance and detail when providing a summary, but it does feel slightly misleading.

<u>Author's Response:</u>

We have revised lines 367–370 to explicitly clarify that the regions referred to are based on general adult population data. We have also removed London from the list of highest Section 42 enquiry regions, consistent with our correction in the results section, where the data show that London ranks below Yorkshire and the Humber and the North East for Section 42 enquiries in the general population.

Among the general adult population, regions such as the South East and North West reported the highest safeguarding activity, both in terms of overall concerns and Section 42 enquiries, which may indicate greater underlying need but could also reflect differences in reporting practices and system responsiveness. Conversely, areas with lower reported concerns, such as the East Midlands and South West, may benefit from targeted awareness campaigns and support to strengthen detection and early intervention. For people living with dementia specifically, Yorkshire and the Humber, the East of England, and the North West recorded the highest safeguarding activity, while London, the South East, and the South West recorded the lowest, underscoring the importance of dementia-specific safeguarding strategies.

<u>Comments from 2nd Reviewer #14:</u>

Reviewer comment: Figures – you say in your response to review comments that you have made changes to the Figures (e.g. category ranges), but I cannot see all of these in the version I have. E.g. Figure 4C still has the dark blue band as having the range '5-5', and Figures 4A, 4B and 4C all still have overlapping intervals such as '10-35', '35-125'.

<u>Author's Response:</u>

We have ensured that all figures have been regenerated with non-overlapping interval boundaries (e.g., '10–34' and '35–124' rather than '10–35' and '35–125') and that the erroneous '5–5' range in

Figure 4C has been corrected to '1–5'. The updated figures are submitted as separate files alongside this revision.
