# Supplementary material for: Geographical and temporal distribution of safeguarding concerns among older adults with dementia in England: a population-based repeated cross-sectional study
Source: BMJ Open. 2026 Aug 26;16(8):e110575. doi: 10.1136/bmjopen-2025-110575 (PMC13536066; doi:10.1136/bmjopen-2025-110575)
Supplement: Supplementary file 2 [file bmjopen-16-8-s001.docx]

**Supplementary Table 1** Predicted number of people living with dementia having Safeguarding Concerns by areas, sex and age groups 2021-22

| **Region** | **Age** | **Males** | | | **Females** | | |
| --- | --- | --- | --- | --- | --- | --- | --- |
|  |  | **Concerns** | **Section 42 Enquiries** | **Other Enquiries** | **Concerns** | **Section 42 Enquiries** | **Other Enquiries** |
| England | Total | 7503 | 3587 | 112 | 12607 | 6028 | 188 |
|  | 0-29 | 1 | 1 | 0 | 2 | 1 | 0 |
|  | 30-34 | 2 | 1 | 0 | 1 | 1 | 0 |
|  | 35-39 | 3 | 1 | 0 | 3 | 1 | 0 |
|  | 40-44 | 6 | 3 | 0 | 5 | 2 | 0 |
|  | 45-49 | 12 | 6 | 0 | 9 | 4 | 0 |
|  | 50-54 | 35 | 17 | 1 | 31 | 15 | 0 |
|  | 55-59 | 89 | 43 | 1 | 90 | 43 | 1 |
|  | 60-64 | 204 | 97 | 3 | 180 | 86 | 3 |
|  | 65-69 | 378 | 181 | 6 | 349 | 167 | 5 |
|  | 70-74 | 786 | 376 | 12 | 864 | 413 | 13 |
|  | 75-79 | 1426 | 682 | 21 | 1858 | 888 | 28 |
|  | 80-84 | 1812 | 866 | 27 | 2774 | 1326 | 41 |
|  | 85-89 | 1713 | 819 | 26 | 3237 | 1548 | 48 |
|  | 90+ | 1035 | 495 | 15 | 3205 | 1532 | 48 |
| North East | Total | 246 | 218 | 6 | 414 | 367 | 9 |
|  | 0- 29 | 0 | 0 | 0 | 0 | 0 | 0 |
|  | 30-34 | 0 | 0 | 0 | 0 | 0 | 0 |
|  | 35-39 | 0 | 0 | 0 | 0 | 0 | 0 |
|  | 40-44 | 0 | 0 | 0 | 0 | 0 | 0 |
|  | 45-49 | 0 | 0 | 0 | 0 | 0 | 0 |
|  | 50-54 | 1 | 1 | 0 | 1 | 1 | 0 |
|  | 55-59 | 3 | 3 | 0 | 3 | 3 | 0 |
|  | 60-64 | 7 | 6 | 0 | 6 | 5 | 0 |
|  | 65-69 | 12 | 11 | 0 | 11 | 10 | 0 |
|  | 70-74 | 26 | 23 | 1 | 28 | 25 | 1 |
|  | 75-79 | 47 | 41 | 1 | 61 | 54 | 1 |
|  | 80-84 | 59 | 53 | 1 | 91 | 81 | 2 |
|  | 85-89 | 56 | 50 | 1 | 106 | 94 | 2 |
|  | 90+ | 34 | 30 | 1 | 105 | 93 | 2 |
| North West | Total | 1339 | 647 | 49 | 2251 | 1088 | 81 |
|  | 0- 29 | 0 | 0 | 0 | 0 | 0 | 0 |
|  | 30-34 | 0 | 0 | 0 | 0 | 0 | 0 |
|  | 35-39 | 1 | 0 | 0 | 0 | 0 | 0 |
|  | 40-44 | 1 | 0 | 0 | 1 | 0 | 0 |
|  | 45-49 | 2 | 1 | 0 | 2 | 1 | 0 |
|  | 50-54 | 6 | 3 | 0 | 5 | 3 | 0 |
|  | 55-59 | 16 | 8 | 1 | 16 | 8 | 1 |
|  | 60-64 | 36 | 18 | 1 | 32 | 16 | 1 |
|  | 65-69 | 68 | 33 | 2 | 62 | 30 | 2 |
|  | 70-74 | 140 | 68 | 5 | 154 | 75 | 6 |
|  | 75-79 | 255 | 123 | 9 | 332 | 160 | 12 |
|  | 80-84 | 323 | 156 | 12 | 495 | 239 | 18 |
|  | 85-89 | 306 | 148 | 11 | 578 | 279 | 21 |
|  | 90+ | 185 | 89 | 7 | 572 | 277 | 21 |
| Yorkshire and The Humber | Total | 1382 | 810 | 19 | 2323 | 1360 | 31 |
|  | 0- 29 | 0 | 0 | 0 | 0 | 0 | 0 |
|  | 30-34 | 0 | 0 | 0 | 0 | 0 | 0 |
|  | 35-39 | 1 | 0 | 0 | 0 | 0 | 0 |
|  | 40-44 | 1 | 1 | 0 | 1 | 0 | 0 |
|  | 45-49 | 2 | 1 | 0 | 2 | 1 | 0 |
|  | 50-54 | 7 | 4 | 0 | 6 | 3 | 0 |
|  | 55-59 | 16 | 10 | 0 | 17 | 10 | 0 |
|  | 60-64 | 38 | 22 | 1 | 33 | 19 | 0 |
|  | 65-69 | 70 | 41 | 1 | 64 | 38 | 1 |
|  | 70-74 | 145 | 85 | 2 | 159 | 93 | 2 |
|  | 75-79 | 263 | 154 | 4 | 342 | 200 | 5 |
|  | 80-84 | 334 | 196 | 5 | 511 | 299 | 7 |
|  | 85-89 | 316 | 185 | 4 | 596 | 349 | 8 |
|  | 90+ | 191 | 112 | 3 | 591 | 346 | 8 |
| East Midlands | Total | 694 | 306 | 2 | 1166 | 514 | 3 |
|  | 0- 29 | 0 | 0 | 0 | 0 | 0 | 0 |
|  | 30-34 | 0 | 0 | 0 | 0 | 0 | 0 |
|  | 35-39 | 0 | 0 | 0 | 0 | 0 | 0 |
|  | 40-44 | 1 | 0 | 0 | 0 | 0 | 0 |
|  | 45-49 | 1 | 0 | 0 | 1 | 0 | 0 |
|  | 50-54 | 3 | 1 | 0 | 3 | 1 | 0 |
|  | 55-59 | 8 | 4 | 0 | 8 | 4 | 0 |
|  | 60-64 | 19 | 8 | 0 | 17 | 7 | 0 |
|  | 65-69 | 35 | 15 | 0 | 32 | 14 | 0 |
|  | 70-74 | 73 | 32 | 0 | 80 | 35 | 0 |
|  | 75-79 | 132 | 58 | 0 | 172 | 76 | 0 |
|  | 80-84 | 168 | 74 | 0 | 257 | 113 | 1 |
|  | 85-89 | 158 | 70 | 0 | 299 | 132 | 1 |
|  | 90+ | 96 | 42 | 0 | 296 | 131 | 1 |
| West Midlands | Total | 705 | 250 | 6 | 1185 | 420 | 9 |
|  | 0- 29 | 0 | 0 | 0 | 0 | 0 | 0 |
|  | 30-34 | 0 | 0 | 0 | 0 | 0 | 0 |
|  | 35-39 | 0 | 0 | 0 | 0 | 0 | 0 |
|  | 40-44 | 1 | 0 | 0 | 0 | 0 | 0 |
|  | 45-49 | 1 | 0 | 0 | 1 | 0 | 0 |
|  | 50-54 | 3 | 1 | 0 | 3 | 1 | 0 |
|  | 55-59 | 8 | 3 | 0 | 8 | 3 | 0 |
|  | 60-64 | 19 | 7 | 0 | 17 | 6 | 0 |
|  | 65-69 | 36 | 13 | 0 | 33 | 12 | 0 |
|  | 70-74 | 74 | 26 | 1 | 81 | 29 | 1 |
|  | 75-79 | 134 | 48 | 1 | 175 | 62 | 1 |
|  | 80-84 | 170 | 60 | 1 | 261 | 92 | 2 |
|  | 85-89 | 161 | 57 | 1 | 304 | 108 | 2 |
|  | 90+ | 97 | 34 | 1 | 301 | 107 | 2 |
| East of England | Total | 1476 | 666 | 0 | 2479 | 1119 | 0 |
|  | 0- 29 | 0 | 0 | 0 | 0 | 0 | 0 |
|  | 30-34 | 0 | 0 | 0 | 0 | 0 | 0 |
|  | 35-39 | 1 | 0 | 0 | 1 | 0 | 0 |
|  | 40-44 | 1 | 0 | 0 | 1 | 0 | 0 |
|  | 45-49 | 2 | 1 | 0 | 2 | 1 | 0 |
|  | 50-54 | 7 | 3 | 0 | 6 | 3 | 0 |
|  | 55-59 | 17 | 8 | 0 | 18 | 8 | 0 |
|  | 60-64 | 40 | 18 | 0 | 35 | 16 | 0 |
|  | 65-69 | 74 | 34 | 0 | 69 | 31 | 0 |
|  | 70-74 | 155 | 70 | 0 | 170 | 77 | 0 |
|  | 75-79 | 280 | 127 | 0 | 365 | 165 | 0 |
|  | 80-84 | 356 | 161 | 0 | 546 | 246 | 0 |
|  | 85-89 | 337 | 152 | 0 | 637 | 287 | 0 |
|  | 90+ | 204 | 92 | 0 | 630 | 285 | 0 |
| London | Total | 438 | 181 | 17 | 737 | 304 | 28 |
|  | 0- 29 | 0 | 0 | 0 | 0 | 0 | 0 |
|  | 30-34 | 0 | 0 | 0 | 0 | 0 | 0 |
|  | 35-39 | 0 | 0 | 0 | 0 | 0 | 0 |
|  | 40-44 | 0 | 0 | 0 | 0 | 0 | 0 |
|  | 45-49 | 1 | 0 | 0 | 1 | 0 | 0 |
|  | 50-54 | 2 | 1 | 0 | 2 | 1 | 0 |
|  | 55-59 | 5 | 2 | 0 | 5 | 2 | 0 |
|  | 60-64 | 12 | 5 | 0 | 11 | 4 | 0 |
|  | 65-69 | 22 | 9 | 1 | 20 | 8 | 1 |
|  | 70-74 | 46 | 19 | 2 | 50 | 21 | 2 |
|  | 75-79 | 83 | 34 | 3 | 109 | 45 | 4 |
|  | 80-84 | 106 | 44 | 4 | 162 | 67 | 6 |
|  | 85-89 | 100 | 41 | 4 | 189 | 78 | 7 |
|  | 90+ | 60 | 25 | 2 | 187 | 77 | 7 |
| South East | Total | 597 | 276 | 6 | 1003 | 464 | 9 |
|  | 0- 29 | 0 | 0 | 0 | 0 | 0 | 0 |
|  | 30-34 | 0 | 0 | 0 | 0 | 0 | 0 |
|  | 35-39 | 0 | 0 | 0 | 0 | 0 | 0 |
|  | 40-44 | 0 | 0 | 0 | 0 | 0 | 0 |
|  | 45-49 | 1 | 0 | 0 | 1 | 0 | 0 |
|  | 50-54 | 3 | 1 | 0 | 2 | 1 | 0 |
|  | 55-59 | 7 | 3 | 0 | 7 | 3 | 0 |
|  | 60-64 | 16 | 8 | 0 | 14 | 7 | 0 |
|  | 65-69 | 30 | 14 | 0 | 28 | 13 | 0 |
|  | 70-74 | 63 | 29 | 1 | 69 | 32 | 1 |
|  | 75-79 | 113 | 52 | 1 | 148 | 68 | 1 |
|  | 80-84 | 144 | 67 | 1 | 221 | 102 | 2 |
|  | 85-89 | 136 | 63 | 1 | 258 | 119 | 2 |
|  | 90+ | 82 | 38 | 1 | 255 | 118 | 2 |
| South West | Total | 625 | 237 | 9 | 1050 | 398 | 16 |
|  | 0- 29 | 0 | 0 | 0 | 0 | 0 | 0 |
|  | 30-34 | 0 | 0 | 0 | 0 | 0 | 0 |
|  | 35-39 | 0 | 0 | 0 | 0 | 0 | 0 |
|  | 40-44 | 0 | 0 | 0 | 0 | 0 | 0 |
|  | 45-49 | 1 | 0 | 0 | 1 | 0 | 0 |
|  | 50-54 | 3 | 1 | 0 | 3 | 1 | 0 |
|  | 55-59 | 7 | 3 | 0 | 8 | 3 | 0 |
|  | 60-64 | 17 | 6 | 0 | 15 | 6 | 0 |
|  | 65-69 | 31 | 12 | 0 | 29 | 11 | 0 |
|  | 70-74 | 65 | 25 | 1 | 72 | 27 | 1 |
|  | 75-79 | 119 | 45 | 2 | 155 | 59 | 2 |
|  | 80-84 | 151 | 57 | 2 | 231 | 88 | 3 |
|  | 85-89 | 143 | 54 | 2 | 270 | 102 | 4 |
|  | 90+ | 86 | 33 | 1 | 267 | 101 | 4 |
